# Antioxidants Prevent Iron Accumulation and Lipid Peroxidation, but Do Not Correct Autophagy Dysfunction or Mitochondrial Bioenergetics in Cellular Models of BPAN

**DOI:** 10.3390/ijms241914576

**Published:** 2023-09-26

**Authors:** Alejandra Suárez-Carrillo, Mónica Álvarez-Córdoba, Ana Romero-González, Marta Talaverón-Rey, Suleva Povea-Cabello, Paula Cilleros-Holgado, Rocío Piñero-Pérez, Diana Reche-López, David Gómez-Fernández, José Manuel Romero-Domínguez, Manuel Munuera-Cabeza, Antonio Díaz, Susana González-Granero, José Manuel García-Verdugo, José A. Sánchez-Alcázar

**Affiliations:** 1 Centro Andaluz de Biología del Desarrollo, ABD-CSIC-Universidad Pablo de Olavide, 41013 Sevilla, Spain; asuacar1@alu.upo.es (A.S.-C.); malvcor@upo.es (M.Á.-C.); aromgon1@upo.es (A.R.-G.); mtalrey1@alu.upo.es (M.T.-R.); spovcab@upo.es (S.P.-C.); pcilhol@alu.upo.es (P.C.-H.); rpieper@alu.upo.es (R.P.-P.); dreclop@alu.upo.es (D.R.-L.); dgomfer1@acu.upo.es (D.G.-F.); jmromdom@upo.es (J.M.R.-D.); mmuncab@upo.es (M.M.-C.); 2 Department of Developmental and Molecular Biology, Albert Einstein College of Medicine, New York, NY 10461, USA; antonio.diazcarretero@einsteinmed.edu; 3 Institute for Aging Studies, Albert Einstein College of Medicine, New York, NY 10461, USA; 4 Laboratory of Comparative Neurobiology, Cavanilles Institute of Biodiversity and Evolutionary Biology, University of Valencia and CIBERNED-ISCIII, 46100 Valencia, Spain; susana.gonzalez@uv.es (S.G.-G.); j.manuel.garcia@uv.es (J.M.G.-V.)

**Keywords:** BPAN, WDR45, antioxidants, autophagy, iron accumulation

## Abstract

Neurodegeneration with brain iron accumulation (NBIA) is a group of rare neurogenetic disorders frequently associated with iron accumulation in the basal nuclei of the brain. Among NBIA subtypes, β-propeller protein-associated neurodegeneration (BPAN) is associated with mutations in the autophagy gene *WDR45*. The aim of this study was to demonstrate the autophagic defects and secondary pathological consequences in cellular models derived from two patients harboring *WDR45* mutations. Both protein and mRNA expression levels of WDR45 were decreased in patient-derived fibroblasts. In addition, the increase of LC3B upon treatments with autophagy inducers or inhibitors was lower in mutant cells compared to control cells, suggesting decreased autophagosome formation and impaired autophagic flux. A transmission electron microscopy (TEM) analysis showed mitochondrial vacuolization associated with the accumulation of lipofuscin-like aggregates containing undegraded material. Autophagy dysregulation was also associated with iron accumulation and lipid peroxidation. In addition, mutant fibroblasts showed altered mitochondrial bioenergetics. Antioxidants such as pantothenate, vitamin E and α-lipoic prevented lipid peroxidation and iron accumulation. However, antioxidants were not able to correct the expression levels of WDR45, neither the autophagy defect nor cell bioenergetics. Our study demonstrated that *WDR45* mutations in BPAN cellular models impaired autophagy, iron metabolism and cell bioenergetics. Antioxidants partially improved cell physiopathology; however, autophagy and cell bioenergetics remained affected.

## 1. Introduction

Neurodegeneration with brain iron accumulation (NBIA) is a clinically and genetically heterogeneous group of neurodegenerative diseases characterized by the abnormal accumulation of brain iron and the progressive degeneration of the nervous system. One of the recently identified subtypes of NBIA is β-propeller protein-associated neurodegeneration (BPAN), also known as neurodegeneration with brain iron accumulation type 5, a rare neurodevelopmental and neurodegenerative disorder [1].

Children affected by BPAN commonly present with developmental delay, seizures and sleep disorders [2,3,4]. Later in life, individuals typically develop dementia and parkinsonism [5]. Affected males have been rarely reported in the literature, as this disorder was first considered to be embryonic lethal in males [6,7]. Despite inheriting two X-chromosomes, females can also be affected because X-chromosome inactivation enables only one of two X-chromosomes to be expressed per cell. Recently, a broader phenotypic spectrum with less severe outcomes has been identified as next generation sequencing and has become more accessible [3]. This suggests that pathogenic variants in *WDR45* may encompass subtypes of distinct disorders or that children with BPAN exist on a spectrum of phenotypes. It is difficult to diagnose BPAN based on clinical features [8]. Currently, the diagnosis is often established after whole-exome sequencing.

BPAN is frequently caused by de novo pathogenic variants in *WDR45* (WD repeat domain 45), a gene with X-linked dominant inheritance encoding a β-propeller protein involved in lysosomal autophagy and endoplasmic reticulum homeostasis [9,10]. These heterozygous mutations involve both missense and truncation mutations that lead to the loss of WDR45 function [3,7].

WDR45/WIPI4 is a WD-repeat β-propeller protein that belongs to the WIPI (WD repeat domain, phosphoinositide interacting) family. The precise cellular function of WDR45 is still largely unknown, but deletions or conventional variants in *WDR45* can lead to macroautophagy/autophagy defects, malfunctioning mitochondria, endoplasmic reticulum stress and unbalanced iron homeostasis [11]

WDR45 regulates autophagy, an evolutionarily conserved mechanism of the degradation and recycling of dysfunctional cellular components [12]. WDR45 deficiency in BPAN patients and cellular animal models may result in defects in autophagic flux [13,14]. However, how WDR45 deficiency leads to brain iron overload remains unclear. The upregulation of toxic iron in the fibroblasts of two BPAN-affected patients [15] suggests that alterations in iron homeostasis are associated with WDR45 deficiency. Furthermore, reports on fibroblasts and pluripotent stem cell-derived neurons from a female patient with BPAN have shown elevated iron levels and mitochondrial and lysosomal dysfunction [16].

In *WDR45*-KO cells, it has been demonstrated that the loss of WDR45 led to defects in ferritinophagy [17], a form of autophagy that degrades the iron storage protein ferritin, contributing to iron accumulation in WDR45-KO cells. Iron accumulation was also detected in the mitochondria, which was accompanied by impaired mitochondrial respiration, elevated reactive oxygen species (ROS) and increased cell death [17].

Ferritinophagy is one of the mechanisms that could link the roles of WDR45 in autophagy to iron homeostasis. Ferritinophagy is the process through which the intracellular iron storage protein ferritin is sequestered within autophagosomes and delivered to lysosomes for degradation [18]. This process is crucial in liberating iron from ferritin and thus in maintaining cellular iron homeostasis [18]. Because of the role of WDR45 in autophagy, it is plausible that WDR45 deficiency promotes defects in ferritinophagy, which may contribute to iron accumulation [17,19,20]. However, the molecular mechanisms that link alterations in autophagy function to changes in iron levels by WDR45 deficiency remain unknown.

Rodent models further reveal the critical roles of WDR45 in neuronal development and survival. Neuron-specific wdr45-knockout (KO) mice display poor motor coordination, learning and memory defects and extensive axon swelling [14]. The primary neuronal cells in these mice show defects in autophagic flux, with the accumulation of ubiquitin-positive aggregates in both neurons and axons [14]. In addition, constitutive wdr45-KO mice display cognitive impairments, abnormal synaptic transmission and lesions in several brain regions [10]. The defect in autophagy caused by WDR45 deficiency leads to elevated endoplasmic reticulum (ER) stress and neuronal apoptosis [16], which is consistent with neurodegeneration found in BPAN patients.

Iron is an essential trace element required by almost all living organisms [21]. As the most metabolically active organ in the body, the brain requires iron for oxidative metabolism, myelination, mitochondrial energy generation and the biosynthesis of neurotransmitters [22]. However, excess iron becomes toxic because of the generation of ROS through the Fenton reaction, resulting in oxidative stress and directly damaging DNA, lipids and proteins [23]. Therefore, the brain requires precise iron homeostasis to guard against iron accumulation while providing the optimal level of this nutrient, which is essential for its development and functions. Recently, our group has proposed that lipid peroxidation and iron accumulation are interconnected pathological processes that reinforce each other and may aggravate and accelerate the progression of neurodegenerative diseases [24].

This study aimed to examine autophagic defects, iron accumulation, lipid peroxidation and bioenergetic defects in WDR45-mutant cellular models from two patients with BPAN. In addition, we evaluated the therapeutic effect of antioxidants such as pantothenate, vitamin E and α-lipoic acid on pathological alterations.

## 2. Results

### 2.1. WDR45 Deficiency Leads to Autophagy Dysfunction in BPAN Cells

**Figure 1:**
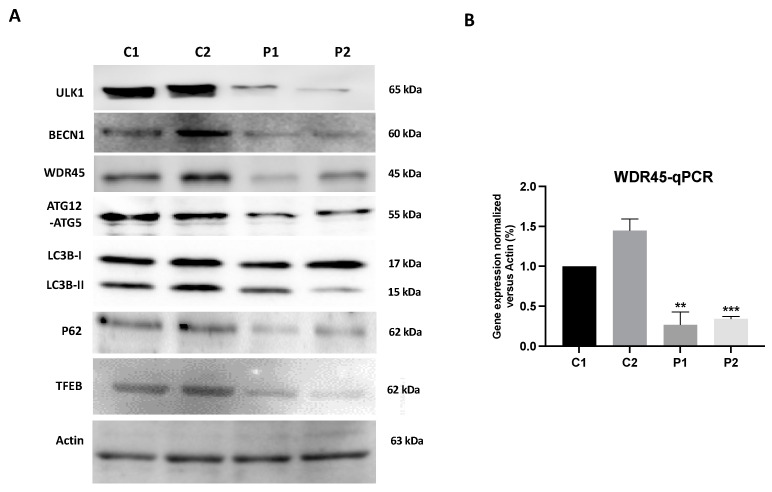
Expression levels of autophagic proteins in BPAN fibroblasts. (**A**) Immunoblotting analysis of cellular extracts from controls (C1 and C2) and BPAN patient cell lines (P1 and P2). Protein extracts (50 μg) were separated on a SDS polyacrylamide gel and immunostained with antibodies against ULK1, BECN1, WDR45, ATG12-ATG5, LC3B, P62 and TFEB. Actin was used as a loading control. (**B**) WDR45 gene expression. WDR45 transcripts were quantified by RT-qPCR. Data represent the mean ± SD of three separate experiments. Results were normalized to actin and referred to C1. ** *p* < 0.005, *** *p* < 0.0005 between BPAN and control cells.

First, we analyzed the WDR45 expression levels in fibroblast cell lines derived from two BPAN patients and two healthy subjects. As shown by the Western blot analysis in Figure 1A and Figure S1, WDR45 expression levels (band around 45 kDa) were markedly reduced in patients P1 and P2, while normal levels were present in control fibroblasts. The low protein expression levels of WDR45 were also associated with a reduction in WDR45 transcript levels, suggesting a decrease in *WDR45* gene expression (Figure 1B).

The reduced expression of the WDR45 protein was accompanied by a decrease in the expression levels of several autophagic proteins such as ULK1 and BECN1, ATG12-ATG5, p62 and LC3B-II (Figure 1A and Figure S1). Low expression levels of WDR45 and LC3B in BPAN cells were also confirmed by immunofluorescence microscopy (Supplementary Figures S2 and S3). We also examined the expression of transcription factor EB (TFEB), the main regulator of autophagy and lysosomal biogenesis, which has been implicated in the pathogenesis of neurodegenerative diseases [25]. We found a significant reduction in TFEB expression in mutant cells (Figure 1A and Figure S1). These results suggest a generalized failure of the autophagy machinery.

**Figure 2:**
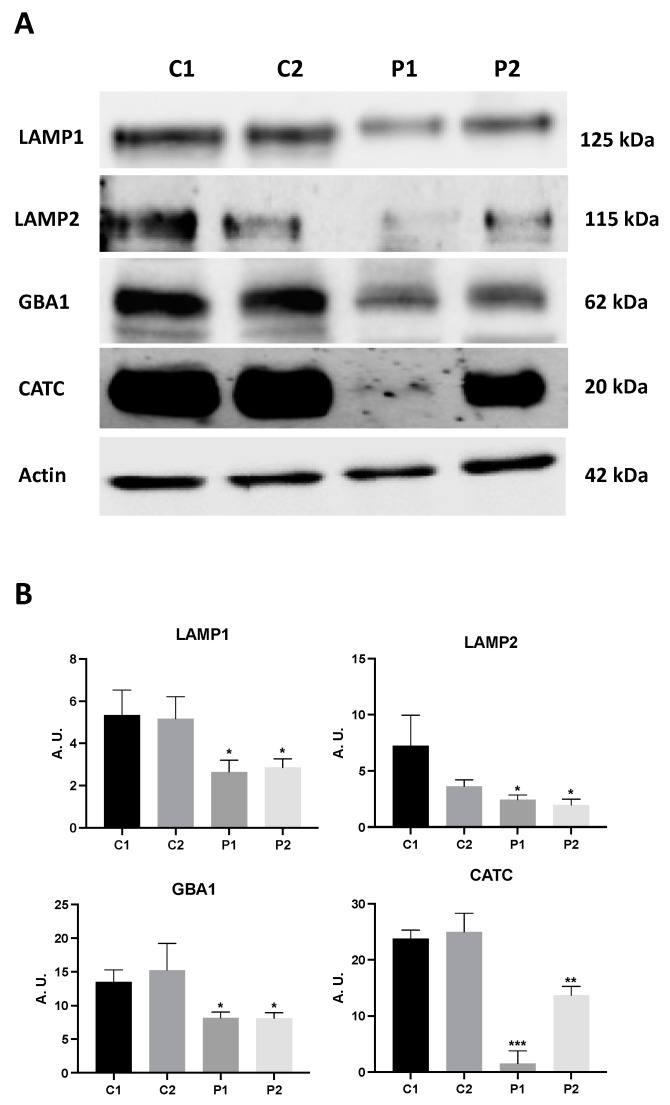
Expression levels of lysosomal proteins in BPAN fibroblasts. (**A**) Immunoblotting analysis of cellular extracts from control (C1 and C2) and BPAN patient cell lines P1 and P2. Protein extracts (50 μg) were separated on a SDS polyacrylamide gel and immunostained with antibodies against LAMP1, LAMP2, GBA1 and CATC. Actin was used as a loading control. (**B**) Densitometry of the Western blotting. Data represent the mean ± SD of three separate experiments. * *p* < 0.05, ** *p* < 0.005, *** *p* < 0.0005 between BPAN and control cells. A.U., arbitrary units.

To investigate how WDR45 deficiency affects the lysosomal compartment, the destination site of the autophagy pathway, we also quantified lysosomal proteins’ expression levels by immunoblotting. Mutant BPAN fibroblasts showed reduced expression levels of lysosome membrane markers, LAMP1 and LAMP2, as well as lysosomal enzymes glucocerebrosidase (GBA1) and catepsin C (CATC) (Figure 2A,B). The deficient lysosomal compartment in BPAN cells was also confirmed by Lysotracker staining (Supplementary Figure S4). Thus, Lysotracker staining was significantly reduced in mutant BPAN cells (P1 and P2) compared to control cells. All together, these results suggest a severe alteration in the autophagic/lysosomal machinery in BPAN fibroblasts.

Consistent with the presence of autophagy dysfunction in mutant cells, serum deprivation did not promote the accumulation of LC3B in BPAN cells (P1 and P2), indicating a defect in autophagosome formation (Supplementary Figure S5A,B). In contrast, serum deprivation induced autophagy and LC3B accumulation in the control cells, suggesting normal autophagosomes formation.

Autophagic dysfunction was also confirmed using the Tandem Sensor RFP-GFP-LC3B, which combines the ability to monitor the various stages of autophagy (autophagosome, autolysosome) through LC3B protein localization (Supplementary Figure S6A,B). By combining an acid-insensitive RFP with an acid-sensitive GFP, the conversion of an autophagosome (neutral pH, indicated by yellow fluorescence) to an autolysosome (acidic pH, indicated by red fluorescence) can be visualized by monitoring the specific loss of the GFP fluorescence (increase in the ratio of RFP- to GFP-positive puncta) upon acidification of the autophagosome following lysosomal fusion [26]. Control cells transiently expressing RFP-GFP-LC3B retained similar levels of GFP- and RFP-positive vacuoles. In contrast, BPAN cells (P1 and P2) transiently expressing RFP-GFP-LC3B showed a reduced number of both GFP- and RFP-positive vacuoles.

Consistent with this, when autophagy was blocked using the lysosomal inhibitor chloroquine (90 μM, Cq), the levels of LC3B (green and red fluorescence) and autolysosomes (red fluorescence) were significantly elevated in the control fibroblasts, indicating autophagosome formation and accumulation (yellow color). In contrast, BPAN fibroblasts showed a reduced amount of autophagosomes and autolysosomes even after chloroquine (Cq) treatment, indicating impaired autophagy.

### 2.2. WDR45 Mutant Cells Show a Deficient Autophagic Flux

**Figure 3:**
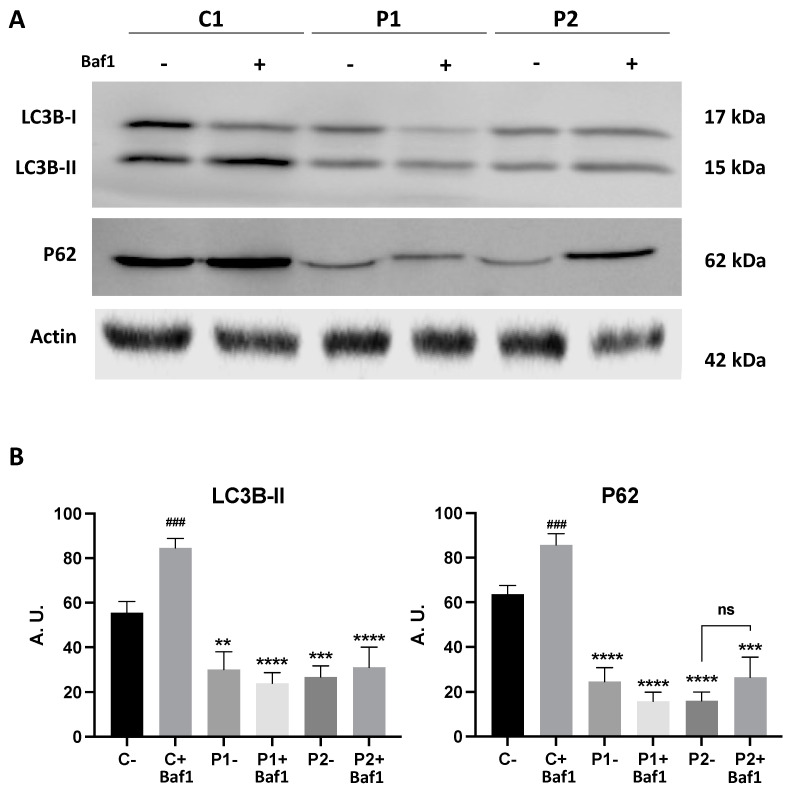
Autophagic flux in control and BPAN cell lines. (**A**) Immunoblotting analysis of cellular extracts from control (C1) and BPAN patient cell lines P1 and P2 untreated (−) and treated (+) with 100 nM of the autophagy inhibitor bafilomycin A1 (Baf1) for 6 h. Blots were immunostained with antibodies against LC3B and P62. Actin was used as a loading control. (**B**) Densitometry of the Western blotting. Data represent the mean ± SD of three separate experiments. ** *p* < 0.005, *** *p* < 0.0005, **** *p* < 0.0001 between BPAN cells and control; ^###^ *p* < 0.0005 between the presence and the absence of Bafilomycin A1. A.U., arbitrary units.

To quantify the autophagy activity, we also monitored autophagy flux using a Western blotting analysis of LC3B-II and P62 in the presence of 100 nM bafilomycin A1 (Baf1) [27], a lysosomal inhibitor. As expected, when autophagy was blocked using the lysosomal inhibitor, the levels of LC3B-II were significantly elevated in the control fibroblasts (Figure 3A,B). However, the increase in LC3B-II after treatment in mutant fibroblasts P1 and in P2 was much lower than in the control cells (Figure 3A,B). These results suggest that autophagic flux was also impaired in BPAN fibroblasts.

### 2.3. WDR45 Mutations Increase Intracellular Iron Levels

**Figure 4:**
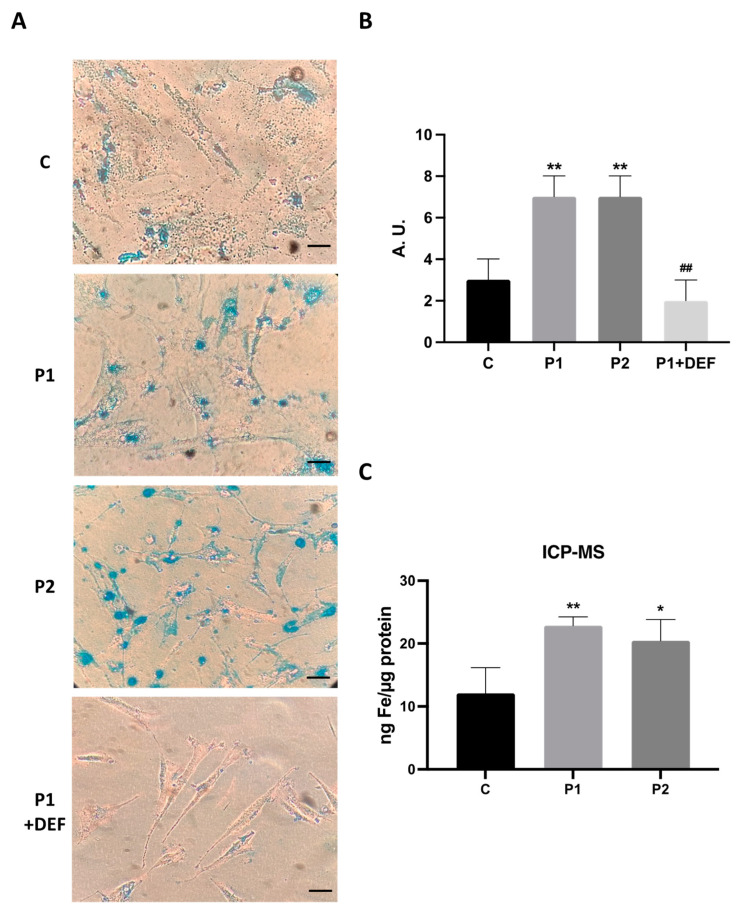
Iron accumulation in BPAN cells. (**A**) Prussian Blue staining of control cells (C1) and BPAN fibroblasts (P1 and P2) cultured in 6-well plates as described in Materials and Methods. The staining solution was removed to visualize the stained cells on the bottom of the wells. P1 fibroblasts treated with 100 μM deferiprone (DEF) for 72 h were used as a negative control. Scale bar = 20 μm. (**B**) Quantification of Prussian Blue staining images was analyzed by the Image J (version 1.53t) software. (**C**) Iron content determined by ICP-MS. Total iron content of control and BPAN patients was determined by ICP-MS as described in Materials and Methods. Data represent the mean ± SD of three separate experiments. * *p* < 0.05, ** *p* < 0.005 between BPAN fibroblasts and controls; ^##^ *p* < 0.005 between the presence and the absence of DEF. A.U., arbitrary units.

As an altered iron metabolism is one of the main characteristics of BPAN mutations, we next examined intracellular iron accumulation by Prussian Blue staining in both the control and BPAN fibroblasts P1 and P2 (Figure 4A,B). Iron staining was significantly increased in BPAN mutant cells. P1 fibroblasts treated with 100 μM deferiprone (DEF) [28], an iron chelating drug, were used as a negative control to corroborate the specificity of Prussian Blue staining for iron. To confirm the abnormal cellular iron content in BPAN fibroblasts, we next determined the intracellular iron levels by induced coupled mass spectrometry (ICP-MS). Mutant BPAN fibroblasts P1 and P2 showed a significant increase in total iron content with respect to the control cells (Figure 4C).

To confirm the role of WDR45 in iron metabolism and autophagosome formation, we next performed cDNA complementation assays, in which we introduced a FLAG-tagged human WDR45 cDNA into the control and P1 and P2 mutant fibroblasts. An immunofluorescence analysis using an anti-WDR45 and anti-FLAG antibody showed that P1 and P2 cells expressed low levels of WDR45, and no FLAG signal was detected (Supplementary Figure S7A,B). However, P1 and P2 cells expressing recombinant WDR45 (rWDR45) had higher WDR45 and FLAG signals (Supplementary Figure S7A,B). Both signals showed high colocalization with a Pearson’s correlation coefficient > 0.90. We then measured autophagosomes’ content at basal conditions by Western blotting and iron overload by Prussian Blue staining (Supplementary Figure S8). The expression of recombinant WDR45 in P1 and P2 cells restored the autophagosome content (LC3-II expression levels) at basal conditions (Supplementary Figure S8A,B). Likewise, the expression of WDR45 significantly eliminated iron accumulation in mutant cells (Supplementary Figure S8C,D). Thus, these data demonstrate a direct link between the expression of WDR45 and autophagosome content and intracellular iron levels.

### 2.4. WDR45 Mutant Cells Accumulate Aberrant Lipofuscin-like Aggregates

**Figure 5:**
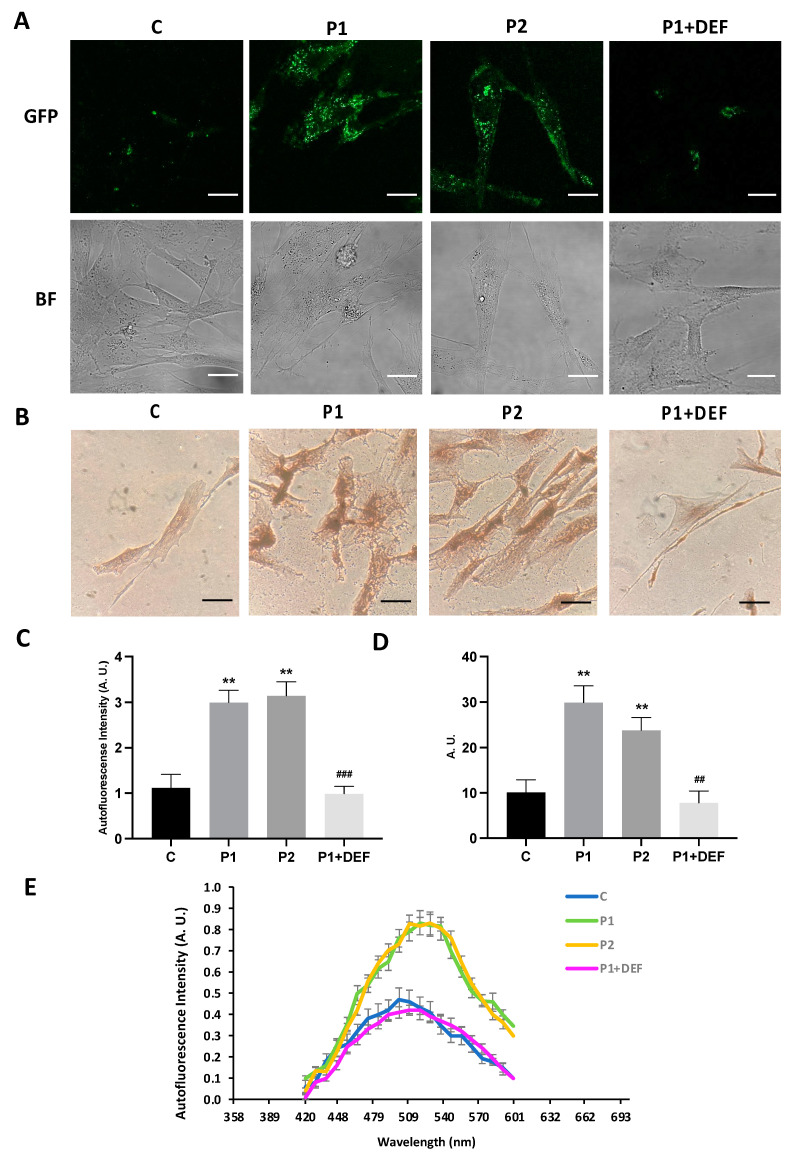
Lipofuscin-like aggregates in BPAN fibroblasts. (**A**) Representative autofluorescence and bright field (BF) images of control (C1) and BPAN fibroblasts (P1 and P2) untreated and treated with 100 μM deferiprone (DEF) for 72 h. Scale Bar = 20 μm. (**B**) Sudan Black staining of control and BPAN fibroblasts. (**C**) Quantification of autofluorescence by image analysis using the Fiji (version 2.9.0) software. (**D**) Quantification of Sudan Black BPAN cells and control (C1). (**E**) The autofluorescence spectra of lipofuscin granules measured by confocal laser scanning microscopy (Nikon A1R, Shinagawa, Tokyo, Japan) in control (C1) and BPAN fibroblasts. P1 was untreated and treated with 100 μM deferiprone (DEF). Excitation laser source: 405 nm. The emission spectra were recorded in 20 large lipofuscin granules in 20 cells. Results are expressed as mean ± SD of autofluorescence intensity. ** *p* < 0.005 between control and BPAN fibroblasts; ^##^ *p* < 0.005, ^###^ *p* < 0.0005 between the presence and the absence of DEF. A.U., arbitrary units.

**Figure 6:**
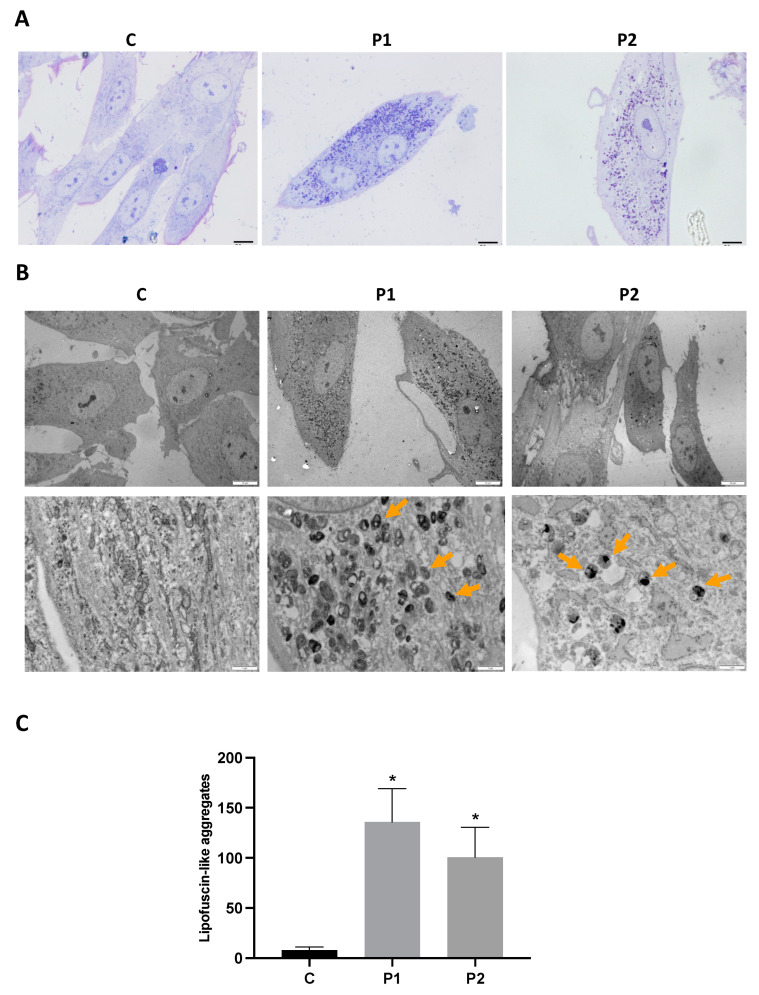
Electron microscopy images of control and P1 and P2 fibroblasts. (**A**) Representative images of semithin sections of 1,5 µm stained with Toluidine Blue and visualized by light microscope. Scale bar = 15 μM. (**B**) Representative electron microscopy images of control (C1) and BPAN fibroblasts (P1 and P2). Top panel scale bar = 10 μm; bottom panel = 1 μm. Orange arrows, lipofuscin-like granules. (**C**) Quantification of lipofuscin-like aggregates. Data represent the mean ± SD of the examination of 50 cells per condition. * *p* < 0.05 between BPAN cell and controls.

As the abnormal accumulation of lipofuscin has been reported in several cell types derived from NBIA patients [28,29], and lipofuscin accumulation can result from lipid peroxidation, a process stimulated by iron [24], we next examined the presence of lipofuscin by an autofluorescence analysis, Sudan Black staining and electron microscopy analysis in the control and BPAN fibroblasts. BPAN mutant cells showed increased autofluorescence and increased Sudan Black staining compared with the control cells, suggesting lipofuscin accumulation (Figure 5). Autofluorescence and Sudan Black staining in patient fibroblasts were significantly reduced after treatment with 100 μM deferiprone (DEF) [28], suggesting that iron was contributing to the accumulation of the autofluorescence and Sudan Black-positive lipofuscin-like material (Figure 5). Furthermore, to confirm the lipofuscin-like characteristic of the aggregates, the fluorescence spectral characteristics of lipofuscin granules in BPAN cells were performed by confocal laser scanning microscopy. Under excitation at 405 nm, lipofuscin granules showed an emission peak at 520–540 nm (Figure 5E). These spectra are consistent with the reported characteristics of lipofuscin granules obtained by Bindewald-Wittich et al. in retinal pigment epithelial cells [30] and Alvárez-Córdoba et al. in pantothenate kinase-associated neurodegeneration (PKAN) cellular models [28]. Increased intracellular lipofuscin-like granules in BPAN fibroblasts were confirmed by TEM examination (Figure 6).

The sequential examination of the mitochondrial morphology in BPAN cells in different stages showed the mitochondrial vacuolization and condensation of some damaged areas of mitochondrial membranes, which eventually were extruded to the cytosol, forming dense lipofuscin-like granules (Supplementary Figures S9–S12). In addition, the rough endoplasmic reticulum (RER) was severely dilated, suggesting ER stress and impaired protein export through the exocytic pathway (Supplementary Figure S13).

### 2.5. WDR45 Deficiency Alters the Expression Levels of Proteins Involved in Iron Metabolism

**Figure 7:**
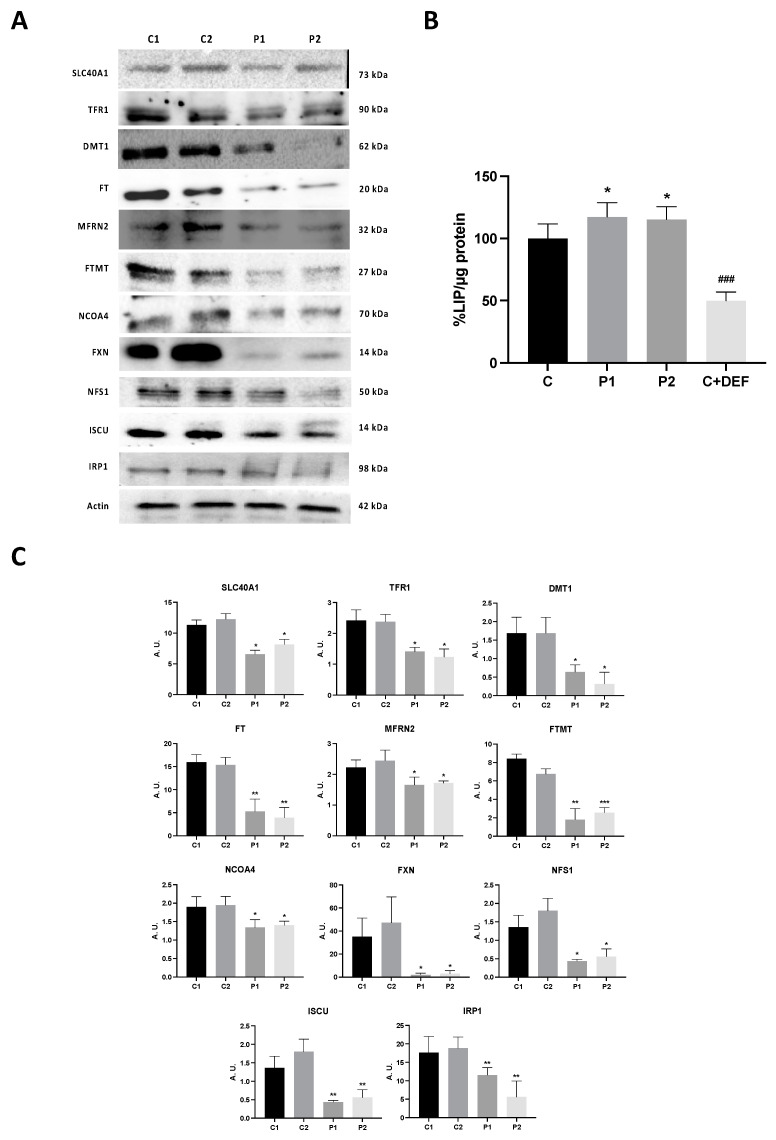
Expression levels of proteins related to iron metabolism. (**A**) Immunoblotting analysis of cellular extracts from controls (C1 and C2) and BPAN patient cell lines P1 and P2. Protein extracts (50 μg) were separated on an SDS polyacrylamide gel and immunostained with antibodies against SLC40A1, TFR1, DMT1, FT, MFRN2, FTMT, NCO4A, FXN, NFS1 and ISCU. Actin was used as a loading control. (**B**) Densitometry of the Western blotting. For control cells (C1 and C2), data are the mean ± SD of the two control cell lines. Data represent the mean ± SD of three separate experiments. * *p* < 0.05, ** *p* < 0.005, *** *p* < 0.0005 between BPAN cells and controls. A.U., arbitrary units. (**C**) Iron labile pool. LIP in control (C1) and BPAN fibroblasts (P1 and P2) assayed as described in Materials and Methods. Control cells treated with 100 μM deferiprone (DEF) for 24 h were used as negative control. For control cells, data are mean ± SD of three control cell lines. * *p* < 0.05 between control and BPAN fibroblasts. ^###^ *p* < 0.0005 between the presence and the absence of DEF. Data represent the mean ± SD of six separate experiments.

As perturbation of the iron metabolism and distribution has been observed in BPAN cells [15], we next assessed the iron metabolism by examining the expression levels of key proteins involved in iron trafficking, storage and regulation, such as SLC40A1 (Ferroportin-1), TFR1, DMT1, FT, MFRN2, FTMT and NCOA4. In addition, the levels of LIP were also quantified by a calcein assay. BPAN mutant fibroblasts showed decreased expression levels of SLC40A1, TFR1, DMT1, FT, MFRN2, FTMT and NCOA4 with respect to the control cells (Figure 7A,C). As an impaired iron metabolism in NBIA fibroblasts has been previously associated with impaired ISC (iron-sulfur cluster) [31,32], we also explored the expression levels of FXN, NFS1 and ISCU in the control and BPAN fibroblasts. The expression levels of FXN, NFS1 and ISCU were dramatically reduced in BPAN fibroblasts, suggesting disorganization of the ISC complex (Figure 7A,C). In addition, we examined the expression level of IRP1 involved in the control of iron metabolism. We observed a significant reduction of IRP1 (Figure 7A,C), suggesting a downregulation in the iron metabolism. Interestingly, LIP was significantly increased in BPAN cells, indicating abnormal iron handling by mutant cells (Figure 7B).

### 2.6. WDR45 Deficiency Impairs Mitochondrial Network and Function

**Figure 8:**
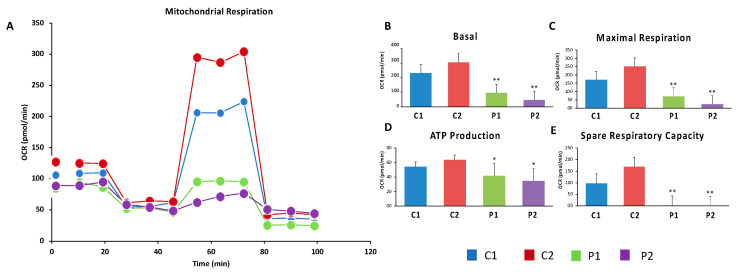
Bioenergetics analysis of control and BPAN fibroblasts. Mitochondrial respiration profile (**A**) was measured with a Seahorse XFe24 analyzer. Parameters of respiratory profile: Basal (**B**) and maximal (**C**) respiration, ATP production (**D**) and spare respiratory capacity (**E**) were determined in controls (C1 and C2) and BPAN fibroblasts (P1 and P2) as described in Materials and Methods. * *p* < 0.05, ** *p* < 0.005 between control and BPAN fibroblasts.

Given that WDR45 is an essential autophagy protein for autophagosome formation and may have an impact on the renewal of mitochondria by mitophagy, we further investigated mitochondrial function. For that purpose, the oxygen consumption rate (OCR) was examined in the control and BPAN fibroblasts (Figure 8A–E). Fibroblasts from patient P1 and P2 showed reduced basal, maximal and spare respiration, as well as decreased mitochondrial ATP production compared to the control cells. This reduced respiratory ability is consistent with the presence of a marked mitochondrial dysfunction.

We next investigated the mitochondrial network morphology after labeling mitochondria with MitotrackerTM Red CMXRos and an examination by fluorescence microscopy. Representative images of mitochondrial morphology are shown in Supplementary Figure S14A,B. A total of 100 cells were quantified for each condition. The mitochondrial network of BPAN fibroblast P1 and P2 showed the presence of depolarized and fragmented mitochondria. The network fragmentation was also evaluated by calculating the percentage of rounded and tubular mitochondria. We observed a marked reduction in the tubular mitochondria of BPAN cells compared to the control cells (Supplementary Figure S14C). As the positive control of mitochondrial membrane potential depolarization, the control cells were treated with 100 μM CCCP (carbonyl cyanide m-chlorophenylhydrazone) for 4 h. The amount of mitochondrial fragmentation was evaluated using the Fiji software. Mitochondrial dysfunction was also confirmed by evaluating the expression levels of mitochondrial proteins (Supplementary Figure S15A,B). Thus, the expression levels of mitochondrial proteins MTND1, COX2, COX4, NDUFS4 and VDAC1 were significantly downregulated in BPAN cells with respect to the control cells.

### 2.7. Mitochondrial Dysfunction Is Associated with Increased Mitochondrial Lipid Peroxidation in BPAN Cells

**Figure 9:**
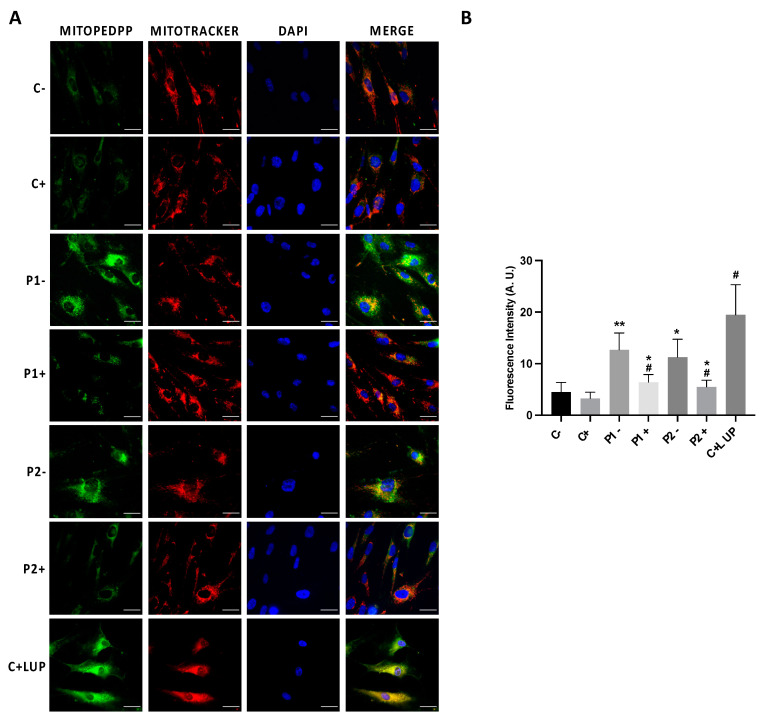
Mitochondrial lipid peroxidation in control and BPAN cells and the effect of antioxidants’ treatment. Control (C1) and BPAN fibroblasts (P1 and P2) were treated (+) with a cocktail of 1 μM α-lipoic acid, 10 μM vitamin E and 5 μM pantothenate for 7 days. (**A**) Levels of mitochondrial lipid peroxidation in untreated (−) and treated (+) control (C1) and BPAN fibroblasts (P1 and P2) assayed by MitoPeDPP staining as described in Materials and Methods. (**B**) MitoPeDPP staining quantification was performed by using the Fiji software. Cells were incubated with MitoTracker^TM^ Deep Red FM to demonstrate that MitoPeDPP signal colocalizes with a mitochondrial marker. Control fibroblasts were treated with 500 μM Luperox (LUP, Tert-butyl hydroperoxide) for 15 min as positive control of lipid peroxidation. Colocalization of both markers MitoPeDPP and MitoTracker was assessed by the DeltaVision (version softWoRx 7.0) software calculating the Pearson correlation coefficient. Pearson correlation coefficient was >0.75 in BPAN fibroblasts and control + LUP. Scale bar = 20 μm. * *p* < 0.05, ** *p* < 0.05 between control and BPAN fibroblasts; ^#^ *p* < 0.05 between the presence and the absence of antioxidants. Data represent the mean ± SD of four separate experiments. A.U., arbitrary units.

As mitochondrial dysfunction can increase ROS generation and consequently may induce lipid peroxidation, we next addressed mitochondrial lipid peroxidation by MITOPeDPP staining. Mutant BPAN cells showed increased levels of mitochondrial lipid peroxidation (Figure 9A,B). A colocalization analysis showed that indeed the MitoPeDPP signal colocalized with MitoTracker™ Deep Red FM, a mitochondrial marker (Pearson correlation coefficient > 0.75). Interestingly, antioxidants such as pantothenate, vitamin E and α-lipoic acid were able to significantly prevent mitochondrial lipid peroxidation (Figure 9A,B).

### 2.8. Antioxidants Prevent Iron Accumulation in BPAN Cells

**Figure 10:**
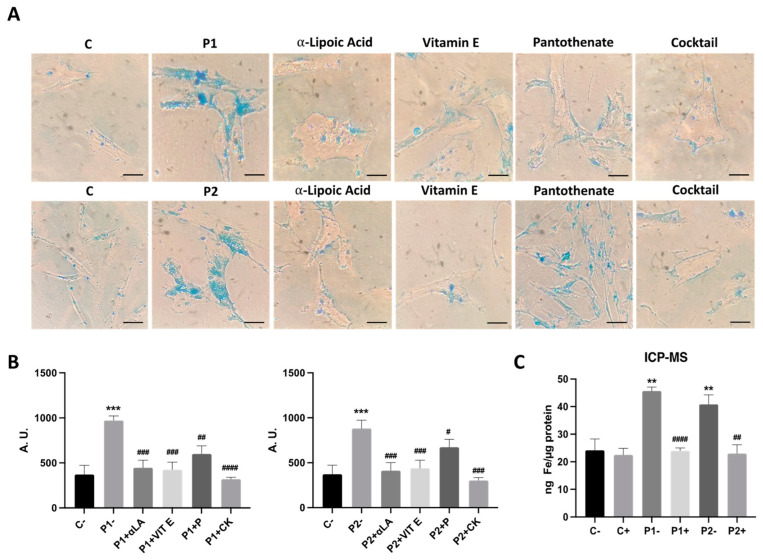
Effect of antioxidants’ treatment on iron accumulation in BPAN cells. (**A**) Control (C1) and BPAN fibroblasts (P1 and P2) were treated with 1 μM α-lipoic acid, 10 μM vitamin E and 5 μM pantothenate individually or in combination for 7 days. Then, cells were stained with Prussian Blue as described in Materials and Methods and examined by bright-field microscopy. Scale bar = 20 μm. (**B**) Quantification of Prussian Blue staining images were analyzed by the Image J software. (**C**) Iron content determined by ICP-MS. Total iron content of untreated (−) and treated (+) control and BPAN patients was determined by ICP-MS as described in Materials and Methods. Data represent the mean ± SD of three separate experiments. ** *p* < 0.005, *** *p* < 0.0005 between control and BPAN fibroblasts; ^#^ *p* < 0.05, ^##^ *p* < 0.005, ^###^ *p* < 0.0005, ^####^ *p* < 0.00005 between the presence and the absence of antioxidants’ cocktail. A.U., arbitrary units.

**Figure 11:**
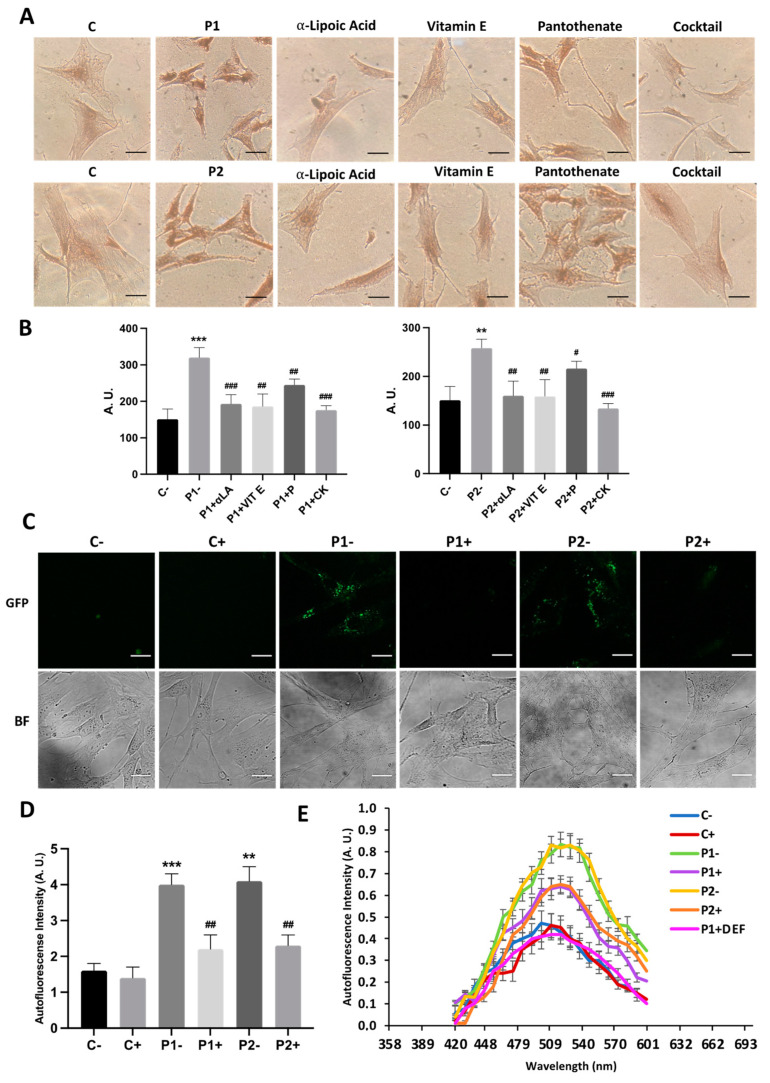
Effect of antioxidant on lipofuscin accumulation addressed by Sudan Black staining and autofluorescence examination. Control (C1) and BPAN fibroblasts (P1 and P2) were treated with 1 μM α-lipoic acid, 10 μM vitamin E and 5 μM pantothenate individually or in combination for 7 days. (**A**) Sudan Black staining. Cells were stained with Sudan Black as described in Materials and Methods and examined by bright-field microscopy. Scale bar = 20 μm. (**B**) Quantification of Sudan Black staining by image analysis using the Image J software. (**C**) Autofluorescence of lipofuscin granules was measured by confocal laser scanning microscopy (Nikon A1R, Shinagawa, Tokyo, Japan) in control (C1) and BPAN fibroblasts (P1 and P2) untreated (−) and treated (+) with 1 μM α-lipoic acid, 10 μM vitamin E and 5 μM pantothenate combined for 7 days. (**D**) Quantification of autofluorescence by image analysis using the Fiji software. (**E**) Emission spectra of untreated (−) and treated (+) cells. Results are expressed as mean ± SD of autofluorescence intensity. ** *p* < 0.005, *** *p* < 0.0005 between control and BPAN fibroblasts; ^#^ *p* < 0.05, ^##^ *p* < 0.005, ^###^ *p* < 0.0005 between the presence and the absence of antioxidants’ cocktail. A.U., arbitrary units.

Given that iron accumulation is associated with lipid peroxidation, and the supplementation with antioxidants prevented both pathological events in several NBIA cellular models [29,33], we next evaluated the ability of individual antioxidants such as pantothenate, vitamin E and α-lipoic acid and the combination of all of them in preventing iron accumulation/lipofuscin accumulation in BPAN fibroblasts. Antioxidants individually or in combination were able to markedly eliminate iron and lipofuscin accumulation in BPAN cells (Figure 10A–C and Figure 11A–E).

### 2.9. Antioxidants Prevent the Accumulation of Lipofuscin-like Aggregates

**Figure 12:**
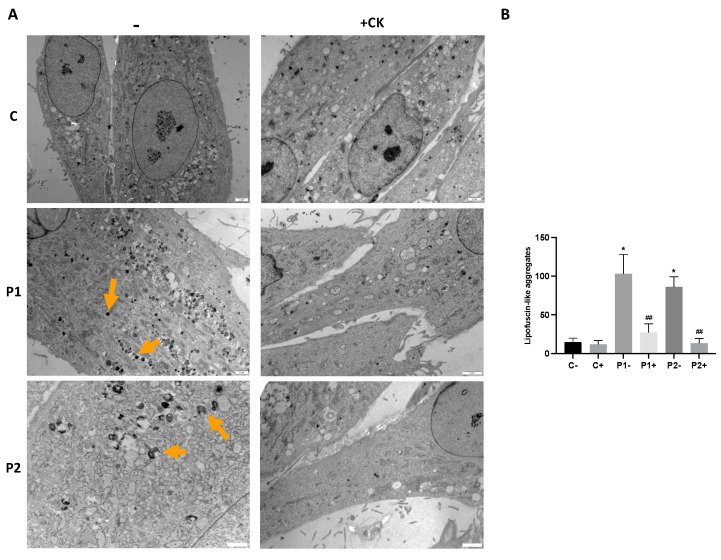
Effect of antioxidants on cell ultrastructure. (**A**) Representative electron microscopy images of control (C1) and BPAN fibroblasts (P1 and P2) treated with antioxidants (1 μM α-lipoic acid, 10 μM vitamin E, 5 μM pantothenate). (**B**) Quantification of lipofuscin-like aggregates (orange arrows) in control and BPAN fibroblasts. Data represent the mean ± SD of the examination of 50 cells per condition. * *p* < 0.05 between control and BPAN fibroblasts; ^##^ *p* < 0.005 between the presence and the absence of antioxidant cocktail. A.U., arbitrary units.

The TEM analysis also revealed that the supplementation with the antioxidant cocktail 5 μM pantothenate, 10 μM vitamin E and 1 μM α-lipoic acid was able to eliminate the accumulation of lipofuscin-like aggregates (Figure 12A,B and Figure S16). In addition, antioxidants were able to significantly reduce RER dilation (Supplementary Figure S17).

### 2.10. Antioxidants Do Not Correct WDR45 Transcripts or Protein Expression Levels in BPAN Fibroblasts

**Figure 13:**
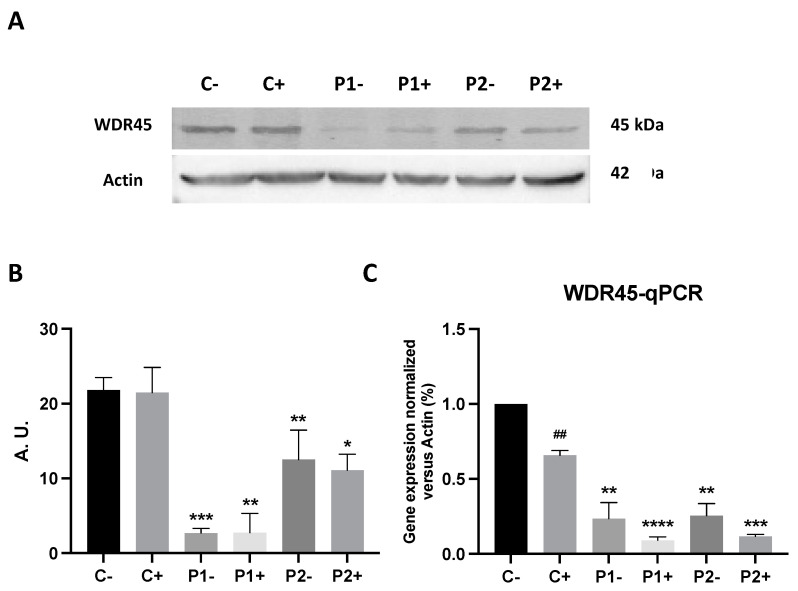
Effect of antioxidants on WDR45 protein and transcripts’ expression levels. (**A**) Immunoblotting analysis of cellular extracts from control (C1) and BPAN patient cell lines P1 and P2 untreated (−) and treated (+) with antioxidants (1 μM α-lipoic acid, 10 μM vitamin E, 5 μM pantothenate) for 7 days. Protein extracts (50 μg) were separated on a SDS polyacrylamide gel and immunostained with antibodies against WDR45. Actin was used as a loading control. (**B**) Densitometry of the Western blotting. For control cells (C1 and C2), data are the mean ± SD of the two control cell lines. (**C**) WDR45 gene expression under antioxidant treatment. WDR45 transcripts were quantified by RT-qPCR. Data represent the mean ± SD of three separated experiments. * *p* < 0.05, ** *p* < 0.005, *** *p* < 0.0005, **** *p* < 0.0001 between BPAN and control fibroblasts. ^##^ *p* < 0.005 between the presence and the absence of antioxidant cocktail. A.U., arbitrary units.

Next, we addressed the effect of the antioxidant treatment on WDR45 gene expression at the transcriptional and protein expression levels. As shown in Figure 13A–C, the combination of the antioxidants pantothenate, vitamin E and α-lipoic acid was not able to increase the transcripts or protein expression levels of WDR45 in BPAN fibroblasts P1 and P2. These results indicate that antioxidants had no effect on WDR45 protein deficiency in BPAN cells.

### 2.11. Antioxidants Do Not Correct Autophagic Failure in BPAN Fibroblasts

**Figure 14:**
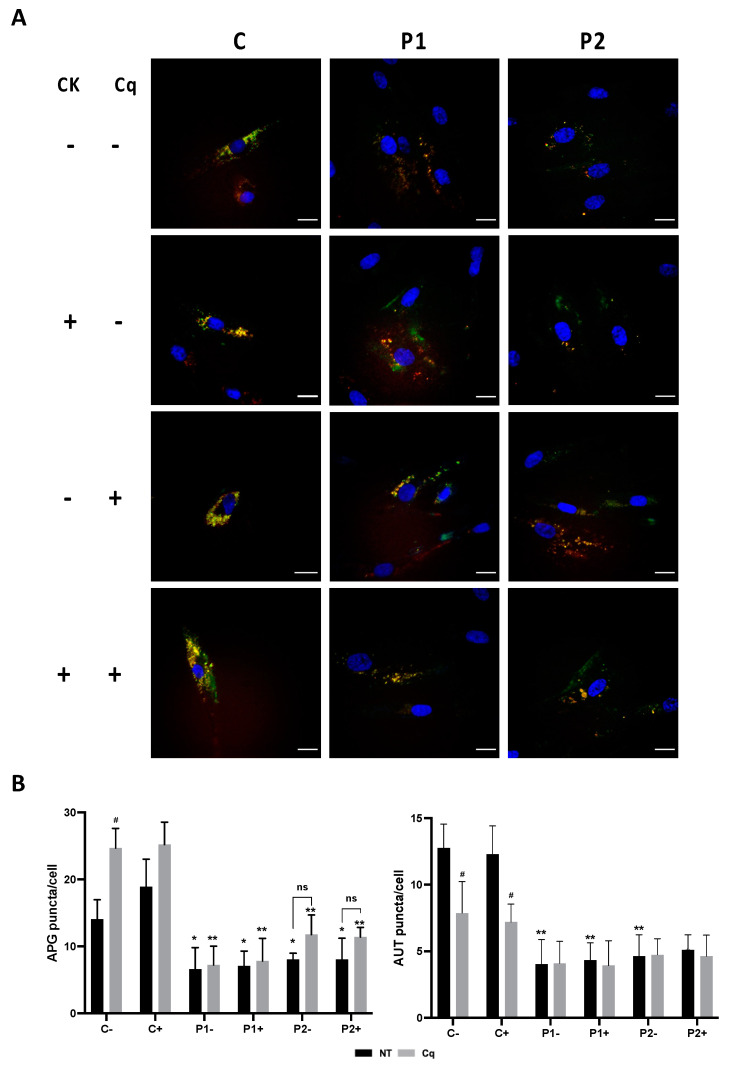
Effect of antioxidants on autophagosome formation in control and BPAN cells. Tandem RFP-GFP-LC3B autophagy sensor was used to determine whether the antioxidant cocktail (CK, 1 μM α-lipoic acid, 10 μM vitamin E and 5 μM pantothenate) was able to increase basal autophagosome formation and after autophagy inhibition by chloroquine (Cq). Control and BPAN cells were untreated (−) and treated (+) with antioxidants for 7 days. Cells were then incubated with either vehicle or 90 μM chloroquine (Cq) for 16 h and imaged. (**A**) Representative fluorescence microscopy images of untreated (−) and treated (+) control and BPAN cells. (**B**) Quantification of RFP and GFP-positive puncta. All images were taken with wide-field fluorescence microscope using a 40X Plan Apo oil objective with standard filter sets for GFP and RFP. Data represent the mean ± SD of three separate experiments. * *p* < 0.05, ** *p* < 0.005 between BPAN cells and controls; ^#^ *p* < 0.01 between the presence and the absence of Cq. APG, autophagosomes; AUT, autolysosomes; ns: not significant.

Furthermore, to assess the effect of antioxidants on the autophagy defect, we used the Tandem Sensor RFP- GFP-LC3B for monitoring autophagosomes and autolysosomes’ formation in the treated and untreated BPAN fibroblasts P1 and P2. The results showed that antioxidants (5 μM pantothenate, 10 μM vitamin E and 1μM α-lipoic acid) were not able to increase autophagosome formation even after chloroquine (Cq) supplementation, suggesting that antioxidants had no positive effect on autophagic failure (Figure 14A,B and Figure S18). Similarly, antioxidants did not correct the reduced content of lysosomes in the BPAN fibroblasts assessed by Lysotracker staining (Supplementary Figure S19).

### 2.12. Antioxidants Do Not Correct Cell Bioenergetics in BPAN Fibroblasts

**Figure 15:**
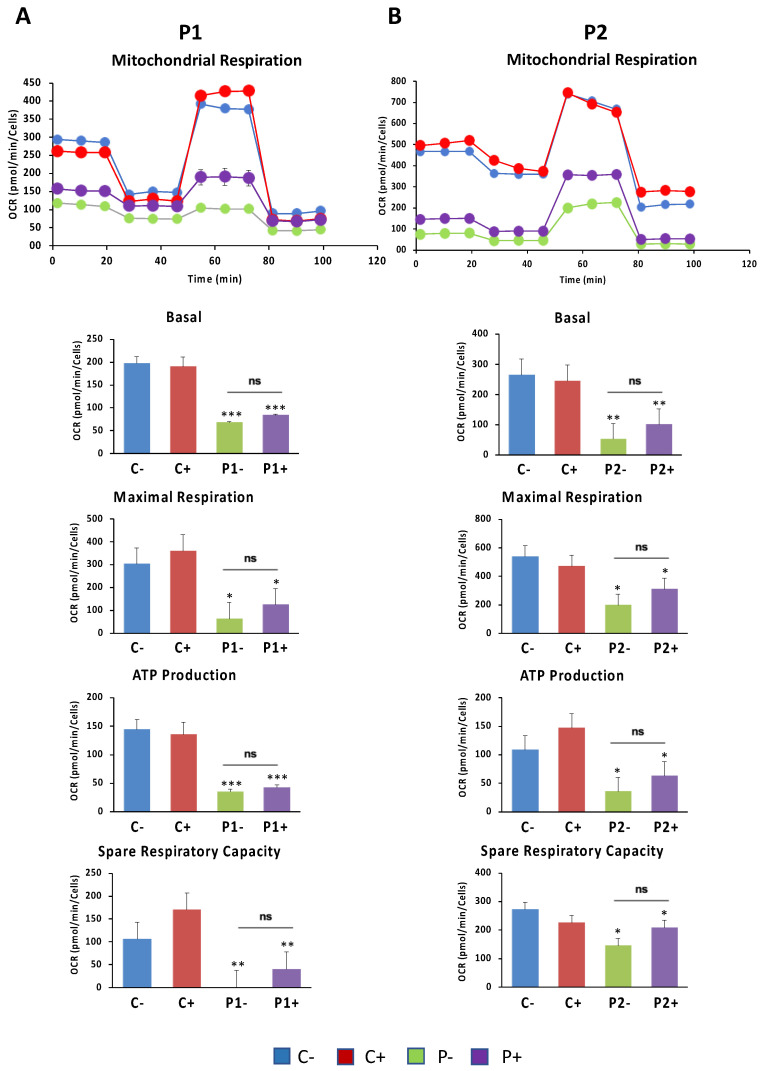
Effect of antioxidant on cell bioenergetics. Control (C1) and BPAN fibroblasts (P1 and P2) were treated (+) with 1 μM α-lipoic acid, 10 μM vitamin E and 5 μM pantothenate for 7 days. Basal, maximal respiration, ATP production and spare respiratory capacity were determined in control (C1) and BPAN fibroblasts P1 (**A**) and P2 (**B**) by using the Seahorse analyzer as described in Materials and Methods. Data represent the mean ± SD of five separate experiments. * *p* < 0.05, ** *p* < 0.005, *** *p* < 0.0005 between control and BPAN fibroblasts; ns: not significant.

We further examined the effect of antioxidants on the mitochondrial function in BPAN fibroblasts using the bioenergetic profile provided by the Seahorse analyzer. Thus, OCR was examined in the control and BPAN fibroblasts P1 and P2 treated and untreated with antioxidants (Figure 15). Antioxidants were not able to correct the reduced basal, maximal and spare respiration, and neither was mitochondrial ATP production in BPAN fibroblasts. We also investigated the effect of antioxidants on mitochondrial network morphology using Mitotracker^TM^ Red CMXRos staining and fluorescence microscopy. Representative images of the effect of the antioxidant cocktail on mitochondrial morphology and polarization are shown in Supplementary Figure S20. The antioxidant cocktail was not able to restore the fragmented mitochondrial network or mitochondrial depolarization in BPAN fibroblasts P1 and P2.

## 3. Discussion

Mutant WDR45 induces diverse cellular consequences including autophagy defects, malfunctioning of mitochondria, mitochondrial lipid peroxidation and iron accumulation, implying that the WDR45 protein functions in diverse pathways that directly or indirectly regulate essential cellular processes [17,19,20,34,35]. However, the casual, functional and temporal sequence of pathological events such as deficient autophagosome formation (with consequences in mitochondrial renewal or ferritinophagy), impaired cell bioenergetics (with consequences in many cellular energy-dependent functions) and altered lipid and iron metabolism (with consequences in cell death by ferroptosis) have not yet been clarified.

In this manuscript, we demonstrated molecular changes in mutant BPAN cells comprising altered autophagy, decreased levels of lysosomal proteins and enzymes, impaired mitochondrial bioenergetics and network, increased mitochondrial lipid peroxidation and iron accumulation, confirming the pathogenicity of the WDR45 variants. The protein and mRNA expression levels of the WDR45 gene were decreased in patient-derived fibroblasts. The amount of increase in LC3B-II upon treatment with an autophagy inducer or inhibitor was also reduced in mutant cells compared to control cells, suggesting decreased autophagosome formation and impaired autophagic flux. Interestingly, TEM showed mitochondrial vacuolization and the accumulation of lipofuscin-like dense aggregates. The decreased lysosomal membrane and enzymes markers also suggested that besides autophagosome formation, the whole autophagic degradation process is altered in WDR45-mutant fibroblasts. The TEM analysis also showed RER distension, suggesting that vesicular traffic and the transport of proteins trough the exocytosis pathway are altered [36]. Thus, the decreased content of membrane and lysosomal enzymes can be explained by the disruption of protein transport from RER to lysosomes.

In line with this, we confirmed the altered autophagic flux in BPAN cells as previously shown in other patient-derived cell lines [7,16]. Accordingly, decreased LC3B-II levels in the WDR45- mutant patient’s fibroblasts in the presence and absence of bafilomycin A1 indicated impaired autophagosome formation and maturation. These findings were also confirmed by using the Tandem Sensor RFP-GFP-LC3B assay, which identifies autophagic vesicles at different stages. Both autophagosome and autolysosome were significantly reduced in BPAN fibroblasts at basal conditions and after the chloroquine treatment.

Lysosomal enzymes’ deficiency in BPAN cells may inhibit the lysosomal clearance of autophagic cargo [37] and therefore may aggravate autophagy failure. Moreover, the impaired autophagosome formation and the depletion of lysosomal enzymes have also been known to trigger dysfunction of mitochondria (the main cellular storage organelles for iron, besides lysosomes) by inhibiting mitochondrial renewal by mitophagy [38]. Indeed, we here showed the altered mitochondrial bioenergetics and network and decreased mitochondrial protein levels in mutant BPAN fibroblasts indicating disrupted mitochondrial integrity that may be caused by an excess of redox-active iron in mitochondria and the resulting ROS generation and lipid peroxidation [39], and/or by the inhibition of mitophagy [38]. These data support the hypothesis that WDR45 mutations lead to autophagic defects resulting in abnormalities in iron-containing organelles such as mitochondria.

One essential question is how and where iron is accumulated in BPAN fibroblasts. Diaw et al. observed that compared to the increased levels of the ferritinophagy marker Nuclear Coactivator 4 (NCOA4) in the control cells upon iron treatment, patients’ cells revealed unchanged NCOA4 protein levels, indicating disturbed ferritinophagy [19]. They concluded that dysfunctional WDR45 might cause deficits in autophagy-like ferritinophagy and, thereby, contribute to disrupted iron homeostasis and ferroptosis, resulting in neurodegeneration in BPAN. Corroborating this hypothesis, Aring et al. have reported that impaired ferritinophagy contributes to iron accumulation in the WDR45-knockout (KO) SH-SY5Y neuroblastoma cell line [17]. However, our findings indicate that ferritin has decreased expression levels in BPAN cells, suggesting that iron is unlikely to be accumulated in this iron storage protein. Our results show that iron accumulation was dependent on mitochondrial lipid peroxidation because antioxidants prevented an iron overload in BPAN fibroblasts. In addition, BPAN cells also showed increased Sudan Black staining and autofluorescence, and a TEM analysis revealed the presence of lipofuscin-like dense aggregates. When mitochondria age, they are digested by lysosomes during mitophagy [40]. However, if the activity of the autophagy machinery and lysosomes is insufficient, then mitochondria can be degraded into granules of lipofuscin (LF) to eliminate the most damaged parts of the mitochondrion. Antioxidants were able to prevent this process. These studies led us to postulate the alternative hypothesis that iron is accumulated in lipofuscin granules that are likely originated from undegraded dysfunctional mitochondria through a process similar to mitoptosis [41,42]. It seems possible that under conditions when the autophagosome formation fails, and consequently mitophagy cannot recycle dysfunctional mitochondria, mitoptosis-like mechanisms may provide cellular protection from the malfunctioning of damaged mitochondria. Interestingly, antioxidants prevent the formation of lipofuscin-like aggregates but do not have a positive effect on mitochondrial dysfunction or bioenergetic failure. Recently, our group has reported that iron is accumulated in lipofuscin granules in cellular models of pantothenate kinase-associated neurodegeneration (PKAN) and PLA2G6-associated neurodegeneration (PLAN), two NBIA disorders [28,29].

Lipofuscin formation has been explained through different mechanisms. Nowadays, two main hypotheses are postulated: the lysosomal and mitochondrial origin of lipofuscin. The lysosomal hypothesis states that lysosomal lipid peroxidation causes lysosomal dysfunction with increased iron permeability through the lysosomal membrane. Then, intralysosomal iron produces hydroxyl radicals by the Fenton reaction which cause the oxidation of lipids and proteins that accumulate in undegradable aggregates [43,44,45,46]. Lipid peroxidation by-products form lipids and proteins’ adducts, which have autofluorescence properties [46]. In addition, altered secondary lysosomes can fuse with other damaged lysosomes leading to lipofuscin accumulation [29,43,44,45]. The lysosomal origin of lipofuscin formation is also related with its mitochondrial origin because damaged mitochondrial renewal can be impaired by low mitophagy and the release of iron by mitochondria in autolysosomes which trigger more Fenton reactions and lipid peroxidation [43,47]. Mitophagy, a selective form of mitochondrial degradation, eliminates ROS-damaged mitochondria, while impaired mitophagy leads to the accumulation of damaged proteins and lipids in mitochondria [48]. The Mitochondrial-Lysosomal Axis Theory, hypothesized by Brunk U.T. and Terman A. in 2002, explains the interconnections between both lipofuscinogenesis origins [49,50]. Lipofuscin formation can be also produced by impaired mitochondrial fission without alteration of the autophagosome–lysosome system [45,51]. The mitochondrial origin of lipofuscin is based on the fact that these organelles contain the main reserve of cellular iron [52], which can contribute to mitochondrial lipid peroxidation and the damage of mitochondrial integrity and function. In turn, lipid peroxidation by-products alter the mitochondrial membranes and facilitate lipofuscin formation [53]. The literature describes different mechanisms of mitochondrial lipofuscinogenesis as a result of lipid peroxidation, without lysosomal participation [51].

WDR45 deficiency also leads to an altered mitochondrial metabolism, and enhanced ROS production, thus potentially contributing to neuronal cell death and neurodegeneration [17]. Mitochondria are cellular organelles that, beyond performing many bioenergetic, biosynthetic and regulatory functions, also play a fundamental role in iron metabolism [52]. Most cells take up iron through receptor-mediated endocytosis, and it is transported to the mitochondria for the biosynthesis of essential protein cofactors involved in redox reactions. For this reason, mitochondrial iron levels should be tightly regulated to provide sufficient iron for numerous cellular processes that use iron as a cofactor, including many proteins of the mitochondrial electron transport chain, while avoiding ROS generation by an excess of redox-active iron [39]. Thus, mitochondrial iron accumulation has been implicated in many neurodegenerative diseases [54,55].

In this work, we found that WDR45 deficiency leads to impaired mitochondrial respiration and increased mitochondrial lipid peroxidation. Consistent with mitochondrial dysfunction, we found markedly reduced levels of nDNA and mtDNA-encoded mitochondrial proteins. We hypothesize that WDR45 deficiency leads to impaired renewal of mitochondria, and therefore, the accumulation of oxidized lipids and proteins, which induce mitochondrial dysfunction and increased ROS production. These abnormal mitochondria with increasing levels of oxidized lipid and proteins and iron undergo a process of mitochondrial vacuolization, while damaged mitochondrial components become dense aggregates which are eventually extruded to the cytosol and finally form lipofuscin-like granules.

In addition, lipid peroxidation-derived reactive compounds can affect the stability and expression levels of several transcription factors and may be responsible for the downregulation of many genes involved in essential metabolic processes including autophagy [24]. Thus, the downregulation of genes involved in autophagy (TFEB, WIPI, ATG5, ATG12), lysosomal membrane proteins and lysosomal acidic hydrolases, as well as genes involved in mitochondrial biogenesis and fatty acid metabolism, has been previously described in WDR45 mutant cells [56].

As antioxidants can be effective in blocking the vicious cycle of lipid peroxidation and iron accumulation [24,29], we evaluated the effect of pantothenate, vitamin E and α-lipoic acid on the main pathological alterations in BPAN mutant cells. The antioxidant cocktail was able to significantly eliminate mitochondrial lipid peroxidation and iron overload as well as RER dilation. However, it did not have a significant effect on the autophagy defect or cell bioenergetics. These data suggest that iron accumulation in BPAN cells is caused by lipid peroxidation, and that both alterations are secondary to the main autophagy defect.

The oxidation of mitochondrial lipids with the concomitant lack of mitochondrial renewal by mitophagy due to the lack of autophagosome formation may lead to the formation of iron-rich lipofuscin granules [28]. However, the prevention of mitochondrial lipid peroxidation by antioxidants did not prevent mitochondrial malfunctioning and the bioenergetic failure. Nevertheless, antioxidant supplementation reduced RER dilation, suggesting that oxidative stress and lipid peroxidation are also involved in RER stress in BPAN cells. RER stress has been linked to oxidative stress in the pathophysiology of numerous diseases [57].

Pantothenate, also called pantothenic acid or vitamin B5, is the primary precursor of coenzyme A (CoA), crucial in several cellular processes such as carbohydrate, fat and protein metabolism [58]. Both the direct form (CoA) and the acetylated form (acetyl-CoA) are involved in energy production and respiration, via the citric acid cycle. It is essential for fatty acid synthesis and β-oxidation, and for cholesterol, lipid and sphingolipid biosynthesis, as well as for the production of steroid hormones and neurotransmitters, i.e., acetylcholine [59,60]. Further, acetyl-CoA plays a role in global histone acetylation, modulating gene expression, cell growth and proliferation [61]. Pantothenic acid, due to the increase of CoA, exerts an antioxidative property and enhances glutathione levels and, in animal models, high concentrations protect neurons from radiation damages [62,63,64]. In fact, pantothenate, by stimulating the synthesis of CoA, may facilitate the activation of the fatty acids required for membrane phospholipids’ remodeling and repair by the Land’s cycle [65], and therefore indirectly may prevent lipid peroxidation. In fact, pantothenate supplementation was able to correct lipid peroxidation in cellular models of pantothenate kinase-associated neurodegeneration (PKAN) [28].

Vitamin E, which comprises a family of tocopherols and tocotrienols, has a powerful lipid antioxidant activity that prevents lipid peroxidation [66]. The protective role of vitamin E in the maintenance of neurological health has been established for many years [67]. Furthermore, the potential protective effects of vitamin E supplementation have been suggested in several neurodegenerative diseases, such as Alzheimer’s disease or Friedreich’s Ataxia, among others [66,67]. Vitamin E, a blocker of lipid peroxidation propagation and an efficient ferroptosis inhibitor, has been reported to reduce lipid peroxidation and iron/lipofuscin accumulation and correct mitochondrial dysfunction and the main pathological alterations in PLAN cellular models [29].

In addition, it is well known that α-lipoic acid is one of the most efficient antioxidants [68] due to its good bioavailability, blood–brain barrier crossing ability and lack of toxic effects at therapeutic doses. Many clinical studies proved the beneficial effect of α-lipoic acid in many pathological conditions such as diabetes, atherosclerosis, heart diseases, cataracts and neurodegenerative diseases [69]. In addition, as mentioned before, α-lipoic acid acts as an antioxidant to directly scavenge almost all forms of free radicals (oxygen and nitrogen), chelate transition and heavy metal ions and mediate the recycling of other endogenous antioxidants such as vitamin E, glutathione and ascorbate [70]. In addition, α-lipoic acid has a protective role against lipid peroxidation [71,72]. Furthermore, α-lipoic acid modulates various signaling cascades either by receptor-mediated or non-receptor-mediated processes [68]. Recently, our research group has shown that α-lipoic acid supplementation can improve the pathological alterations in cellular models of PKAN with residual PANK2 expression levels by a mechanism involving the upregulation of PANK2 transcription and enzyme expression levels [73].

## 4. Materials and Methods

### 4.1. Reagents

Monoclonal Anti-Actin antibody, vitamin E, pantothenate, α-lipoic acid, Sudan Black B, Perl’s Prussian Blue, sodium pantothenate, Luperox^®^ DI, deferiprone, carbonyl cyanide m-chlorophenylhydrazone (CCCP), bafilomycin A1, glutaraldehyde 25% Aqueous Solution and trypsin were purchased from Sigma-Aldrich Chemical Co. (St. Louis, MO, USA). MitoTrackerTM Red CMXRos, MitoTrackerTM Deep Red FM, BODIPY^®^ 581/591 C11, anti-ISCU (Iron-Sulfur Cluster Assembly Enzyme), anti-WDR45 (WD repeat domain 45), anti-FXN (Frataxin) Premo™ Autophagy Tandem Sensor RFP-GFP-LC3B Kit, LysoTracker™ Green DND-26, DAPI and Hoechst 3342 were purchased from ThermoScientific™ (Waltham, MA, USA). MitoPeDPP was purchased from Dojindo Molecular Technologies, Inc. (Rockville, MD, USA). Anti-ULK1 (Unc-51-Like Autophagy Activating Kinase 1), anti-BECN1 (Coiled-Coil Myosin-Like BCL2-Interacting Protein), anti-TFEB (Transcription Factor EB), anti-LAMP1 (Lysosomal-Associated Membrane Protein 1), anti-LAMP2 (Lysosomal-Associated Membrane Protein 2), anti-GBA1 (β-glucosidase) and anti-CATC (Cathepsin-C), anti-ferritin light-chain (FT), anti-SLC40A1 (Ferroportin-1), anti-IRP1 (Iron-Responsive Element-Binding Protein 1) and anti-NFS1 (NFS1 Cysteine desulfurase) were purchased from Santa Cruz Biotechnology (Santa Cruz, CA, USA). A cocktail of protease inhibitors (complete cocktail) was purchased from Boehringer Mannheim (Indianapolis, IN, USA). The Immun-Star HRP substrate kit and DC (detergent compatible) protein assay were purchased from Bio-Rad Laboratories Inc. (Hercules, CA, USA).

Anti-P62 (Autophagy Receptor P62) and anti-LC3B (Microtubule-Associated Proteins 1A/1B Light Chain 3B), anti-TFR1 (Transferrin receptor protein 1), anti-NCOA4 (Nuclear receptor coactivator 4), anti-FTMT (Mitochondrial Ferritin), anti-MFRN2 (Mitoferrin-2), Anti-MTND1 (Mitochondrially Encoded NADH:Ubiquinone Oxidoreductase Core Subunit 1), anti-COX2 (Mitochondrially Encoded Cytochrome C Oxidase II), anti-COX4 (Mitochondrially Encoded Cytochrome C Oxidase II), anti-NDUFS4 (NADH:ubiquinone oxidoreductase subunit S4), anti-VDAC1 (Voltage-dependent anion channels) and anti-PGC1α (PPARG Coactivator 1 Alpha) were purchased from Abcam (Cambridge, UK). Anti-ATG12-ATG5 (Autophagy-Related 12- Autophagy-Related 5) was purchased from Cell Signaling Technology (Danvers, MA, USA).

Anti-DMT1 (Divalent Metal Transporter 1) was purchased from EMD Millipore. The XF Mito-stress test kit including oligomycin, carbonyl cyanide ptrifluoromethoxy- phenylhydrazone (FCCP), rotenone and antimycin A was obtained from Seahorse Bioscience Inc. (Billerica, MA, USA), as well as the XFe24 cell culture plates, sensor cartridges (Seahorse XFe FluxPax) and Seahorse XF base medium.

### 4.2. Patient Cell Cultures

We used primary skin fibroblasts from two unaffected subjects of the same age and sex (controls 1, 2) purchased from ATCC and two patients. BPAN patients presented clinical and radiologic evidence compatible with BPAN. One BPAN patient (P1) was a 7-year-old female carrying a heterozygous frame shift mutation c.400C>T (p.Arg134*) that resulted in a truncated WDR45 protein [74], while patient P2 was a 14-year-old female harboring a heterozygous c.182A>C mutation; this variant led to an amino acid change from an asparagine to a threonine at position 61 of the protein (p.Asn61Thr). The in silico prediction SIFT and PolyPhen-2 algorithms estimated that the p.Asn61Thr change could have a deleterious effect [75]. On the other hand, at the same amino acid position, it has been previously described as another pathological variant (p.Asn61Lys) [76].

The control values represented means  ±  SD for two control fibroblast cell lines. Fibroblasts were grown in DMEM (Sigma) supplemented with 10% FBS (Sigma), 100-mg/mL streptomycin, 100-U/mL penicillin and 4-mM l-glutamine (Sigma). All the experiments were performed with fibroblasts’ cell cultures with a passage number < 10.

### 4.3. Cell Transfection with Human WDR45 Plasmid

The FLAG-tagged human WDR45 plasmid (rWDR45) (NM_007075.3) and the anti-DYKDDDDK tag antibody (A00187) were purchased from GenScripts (Piscataway, NJ, USA). Plasmid transfection was performed following the manufacturer’s instructions. Lipofectamine^®^ 2000 was purchased from Thermo Fisher Scientific.

### 4.4. Immunoblotting

Western blotting was performed using standard methods. After the protein transfer, the membrane was incubated with various primary antibodies diluted 1:1000 and then with the corresponding secondary antibody coupled to horseradish peroxidase at a 1:10,000 dilution. Specific protein complexes were identified using the Immun-Star HRP substrate kit (Biorad Laboratories Inc., Hercules, CA, USA).

### 4.5. Quantitative Real-Time PCR

The expression of the WDR45 gene in fibroblasts was analyzed by a real-time quantitative PCR using mRNA extracts. mRNA was extracted by using standard methods and the SYBR Green protocol as a method designed to detect accurate quantification of the gene expression and RT-PCR reactions. WDR45 primers used 5′-TTTACGGTTCCGGGTACTGG-3′ (Forward primer) and 5′-AATTTCAACCTCCACCAGCG-3′ (Reverse primer), amplifying a sequence of 98 nucleotides. Actin was used as a housekeeping control gene and the primers were 5′-AGAGCTACGAGCTGCCTGAC-3′ (Forward primer) and 3′-AGCACTGTGTTGGCGTACAG-5′ (reverse primer).

### 4.6. Inmunofluorescence Microscopy

Immunofluorescence microscopy was performed as described previously by our group [28]. Samples were processed and imaged using a LEICA DMRE fluorescence microscope (Leica Microsystems GmbH, Wetzlar, Germany). A colocalization analysis was performed by the Pearson correlation coefficient using the DeltaVision system (Applied Precision; Issaquah, WA, USA) software.

### 4.7. Lysotracker Staining

Lysotracker is an acidotropic dye that stains cellular acidic compartments, including lysosomes and autolysosomes. For the staining of lysosomes, 75 nM LysoTracker™ Green DND-26 was added for 30 min (Thermo Fisher Scientific). The specimens were examined with a wide-field fluorescence microscope using 40x objective. The number of Lysotracker-positive puncta was analyzed in 100 individual cells. For the fluorescence intensity measurements, mean fluorescence was analyzed in randomly selected areas using the DeltaVision software (Applied Precision; Issaquah, WA, USA).

### 4.8. Tandem Sensor RFP-GFP-LC3B

Autophagic flux was monitored using the Premo™ Autophagy Tandem Sensor RFP-GFP-LC3B kit (Thermo Fisher Scientific, Grand Island, NY, USA) following the manufacturer’s instructions. Cells were treated with 10 μL of BacMam reagents containing the RFP-GFP-LC3B construct and were transduced with 40 particles per cell for 16 h. After incubation, BacMam reagents were removed, and the cells were maintained for 48 h in the medium to normalize the autophagy markers’ expression levels. The control cells were treated with 90 μM chloroquine (Component B) for 16 h following the product guide prior to experiment imaging. Hoechst 33342 staining was used to label cell nuclei. Images were analyzed for green fluorescence (LC3B positive autophagosomes) and red fluorescence (autolysosome formation) using a wide-field fluorescence microscope using 40× objective. The colocalization of green and red fluorescence (GFP + RFP) was indicative of neutral pH autophagosomes, and red fluorescence (RFP) was indicative of acidic pH autolysosomes. The number of positive puncta was analyzed in 100 individual cells. Imaging was performed using the DeltaVision software (Applied Precision; Issaquah, WA, USA).

### 4.9. Iron and Lipofuscin Accumulation

Iron overload was evaluated by Prussian Blue staining and quantified in a microplate reader (Polar Star Omega, BMG Labtech, Ortenberg, Germany) and by bright-field microscopy. A quantification analysis was performed by using the Image J software.

Lipofuscin granules were visualized by Sudan Black B (SBB) staining as previously described [77]. The quantification of SSB staining was performed in a microplate reader and by bright-field microscopy. The cell’s autofluorescence was measured by fluorescence microscopy (excitation 366 nm; emission 420–600 nm). Confocal laser scanning microscopy (Nikon A1R, Shinagawa, Tokyo, Japan) was used for obtaining the emission spectra of lipofuscin granules as previously described [28]. The emission spectra were recorded in 20 lipofuscin granules in 20 cells. The iron content in cell extracts was also determined by ICP-MS [78]. Iron concentration was expressed as nmol Fe^2+^/μg protein. Data were presented as means  ±  SD (standard deviation), *n* = 3 in all cases.

### 4.10. Iron Metabolism and Labile Iron Pool

Labile iron pool (LIP) determination was carried out as a slightly modified version of what has been previously described [31]. Briefly, fibroblasts were plated in 96-well plates. Cells were incubated in the medium supplemented with 1 mg/mL BSA and 0.25 μm Calcein-AM at 37 °C for 15 min. After two washes with Hank’s Balanced Salt Solution (HBSS), cells were maintained in HBSS supplemented with 10 mm glucose for 10 min. Basal fluorescence was measured using a Polar Star Omega Microplate Reader at 485 nm (excitation) and 535 nm (emission). Cells were then supplemented with the specific iron chelator Salicyladehyde Isonicotinoyl Hydrazone (0.1 mM) for 15 min. Fluorescence was followed during incubation with the chelator, and when a plateau was reached, that value was considered to be the LIP value. Finally, the results were normalized to the protein content. The control cells were treated with 100 μM deferiprone as a negative control. TFR, DMT1, FT, NCOA4, SCL40A1, FTMT, MFRN2, FXN, NFS1, ISCU and IRP1 protein expression levels were determined by Western blotting.

### 4.11. TEM Analysis

The cells were seeded on 8-well Permanox chamber slides (Nunc, Thermo Scientific) and were subsequently fixed in tempered 3.5% glutaraldehyde in 0.1 M phosphate buffer (PB) for 10 min at 37 °C and 50 min for 4 °C. Cells were postfixed in 2% OsO_4_ for 1 h at room temperature, rinsed, dehydrated and embedded in Durcupan resin (Fluka, Sigma-Aldrich). Semithin sections (1.5 µm) were cut with a diamond knife and stained lightly with 1% toluidine blue. Later, ultra-thin (70 nm) sections of the cells were cut with a diamond knife, stained with lead citrate (Reynolds solution) and examined under a transmission electron microscope (FEI Tecnai G2 Spirit BioTwin) with a Xarosa (20 Megapixel resolution) digital camera using Radius image acquisition software (version 2.1, EMSIS GmbH, Münster, Germany).

### 4.12. Bioenergetic and Oxidative Stress Analysis

The mitochondrial respiratory function of the control and BPAN fibroblasts was measured using the Mito-stress test assay by XF24 extracellular flux analyzer (Seahorse Bioscience). The protocol has been previously described [28]. Basal respiration, maximal respiration, spare respiratory capacity and ATP production were quantified by calculating the oxygen consumption rate (OCR; pmol O_2_/min), normalized to cell number, after the sequential addition of oligomycin, FCCP and rotenone/antimycin A. A minimum of five wells per experimental condition were utilized.

Mitochondrial lipid peroxidation was determined by MitoPeDPP staining following the manufacturer’s instructions [28]. Localization of the MitoPeDPP signal in mitochondria was addressed by a colocalization analysis with MitoTracker™ Deep Red FM (100 nM, 45 min, 37 °C), a mitochondrial dye. A colocalization analysis was performed by the Pearson correlation coefficient using a DeltaVision system (Applied Precision; Issaquah, WA) software.

### 4.13. Mitotracker Staining: Analysis of Mitochondrial Network

Cells were stained with MitoTracker Red CMXRos (100 nM, 45 min, 37 °C) and examined by fluorescence microscopy as previously described [28]. Mitochondrial fragmentation was examined using the Fiji software 2.9.0. The small and rounded mitochondria per cell were calculated in 100 cells in three different experiments.

### 4.14. Statistics

A statistical analysis was routinely performed as formerly described by our research group [27]. We used non-parametric statistics that did not have any distributional assumption in cases when the number of events was small (*n* < 30) [79]. In these cases, multiple groups were compared using a Kruskal–Wallis test. In cases when the number of events was higher (*n* > 30), we applied parametric tests. In these cases, multiple groups were compared using a one-way ANOVA. Statistical analyses were conducted using the GraphPad (version Prism 10.0.2) (GraphPad Software, San Diego, CA, USA). The data were reported as the mean ± SD values or as representative of at least three independent experiments. *p*-values of less than 0.05 were considered significant.

## 5. Conclusions

Altogether our data suggest that dysfunctional WDR45 leads to autophagic defects resulting in disrupted iron recycling/storing and lipid peroxidation, lipid peroxidation in iron-rich organelles, such as mitochondria which cannot be renewed by mitophagy, may lead to the formation of lipofuscin-like aggregates which are extruded from mitochondria through a process similar to mitoptosis. These iron-rich aggregates of oxidized lipids and proteins may lead to lipofuscinogenesis, which in turn increases lipid peroxidation. This vicious cycle reinforces itself and may aggravate and accelerate the progression of neurodegeneration in BPAN disease [24].

Furthermore, we conclude that antioxidants may ameliorate lipid peroxidation and iron overload in BPAN cellular models. However, antioxidants did not have a positive effect on autophagy deficiency or cell bioenergetics. Further investigations are required to reveal the connections among autophagy deficiency, lipid peroxidation and iron accumulation in BPAN.

## Data Availability

Data and materials are available by request.

## Supplementary Materials

The following supporting information can be downloaded at: https://www.mdpi.com/article/10.3390/ijms241914576/s1.
