# Supplementary material for: Antioxidants Prevent Iron Accumulation and Lipid Peroxidation, but Do Not Correct Autophagy Dysfunction or Mitochondrial Bioenergetics in Cellular Models of BPAN
Source: Int J Mol Sci. 2023 Sep 26;24(19):14576. doi: 10.3390/ijms241914576 (PMC11340724; doi:10.3390/ijms241914576)
Supplement: Supplementary file 1 [file ijms-24-14576-s001.zip › Supplementary Figures-finalPDF.pdf]

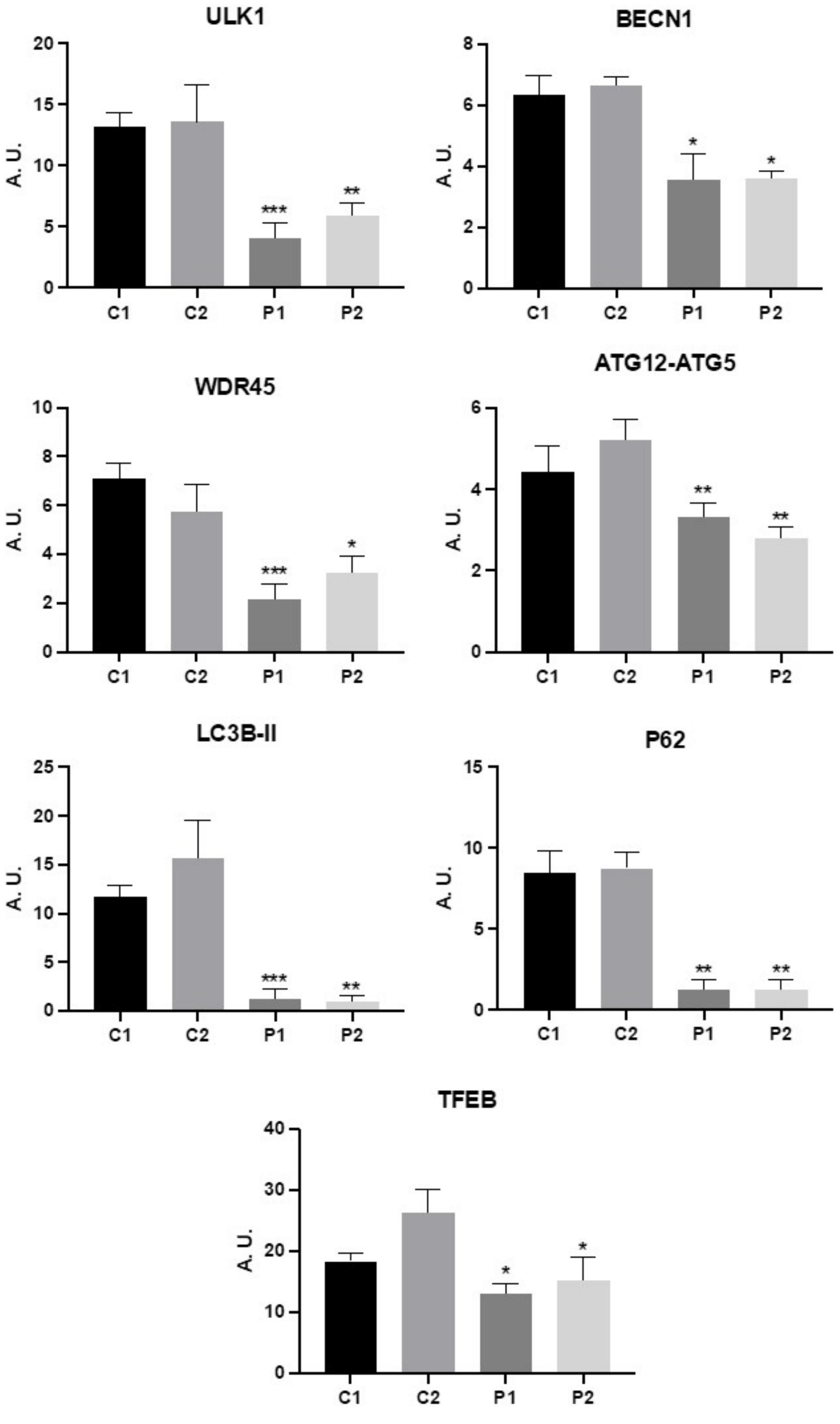

Supplementary Figure S 1. Densitometry of Western blotting of Figure 1. Data represent the mean ± SD of three separate experiments. For control cells (C1 and C2), data are the mean±SD of the two control cell lines. *p<0.05, ** p<0.005, ***p<0.0005 between controls and BPAN fibroblasts.

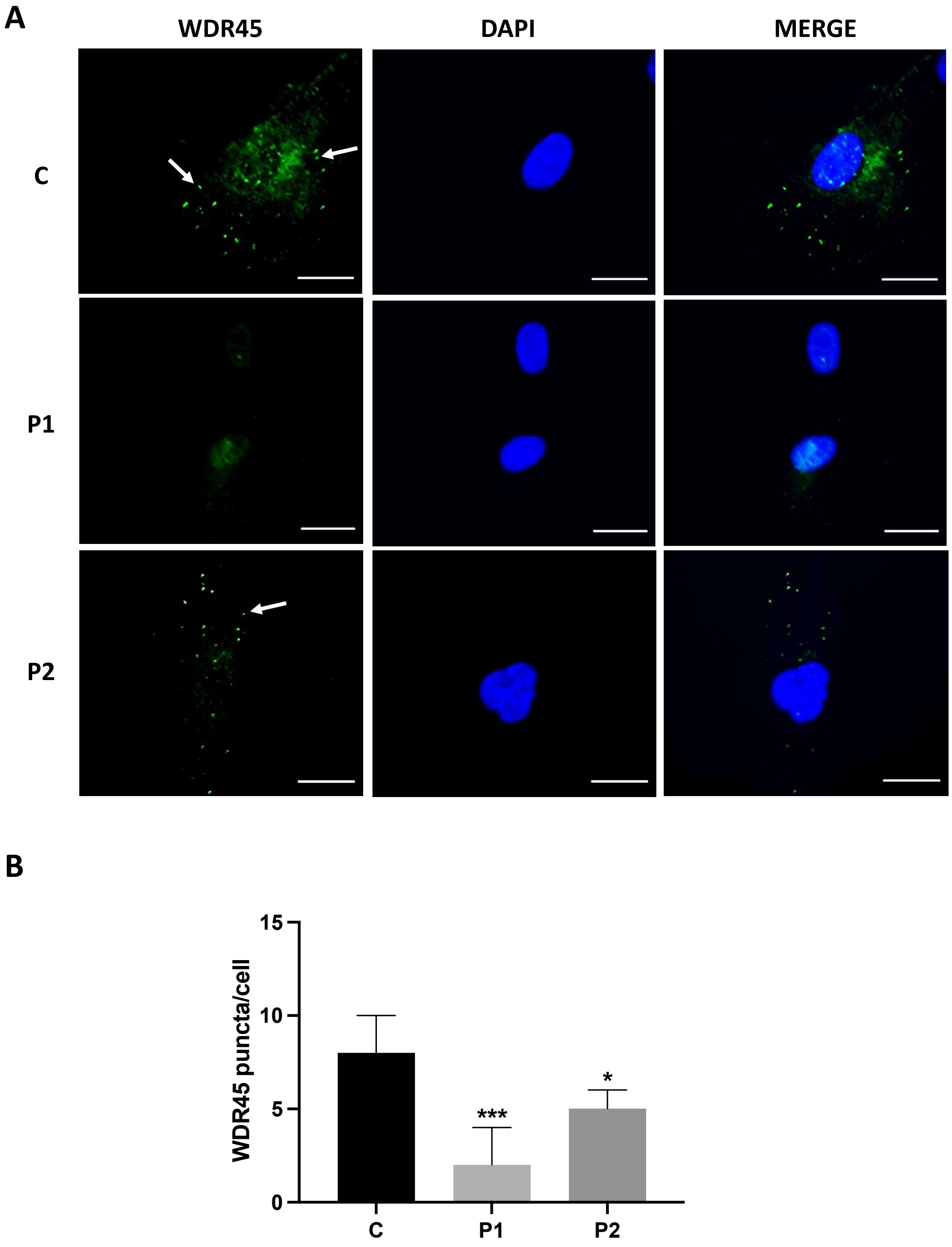

Supplementary Figure S 2. WDR45 expression levels by immnunofluorescence microscopy. (**A**) Control (C) and BPAN cells (P1 and P2) were immunostained against WDR45 and visualized under widefield fluorescence microscope. Nuclei were revealed by DAPI staining. (**B**) Puncta (white arrows) quantification of WDR45 signal. Data represent the mean ± SD of three separate experiments (at least 100 cells for each condition and experiment were analysed). *p<0.05, ** p<0.005 between Control and BPAN fibroblasts. Scale bars=20 µm.

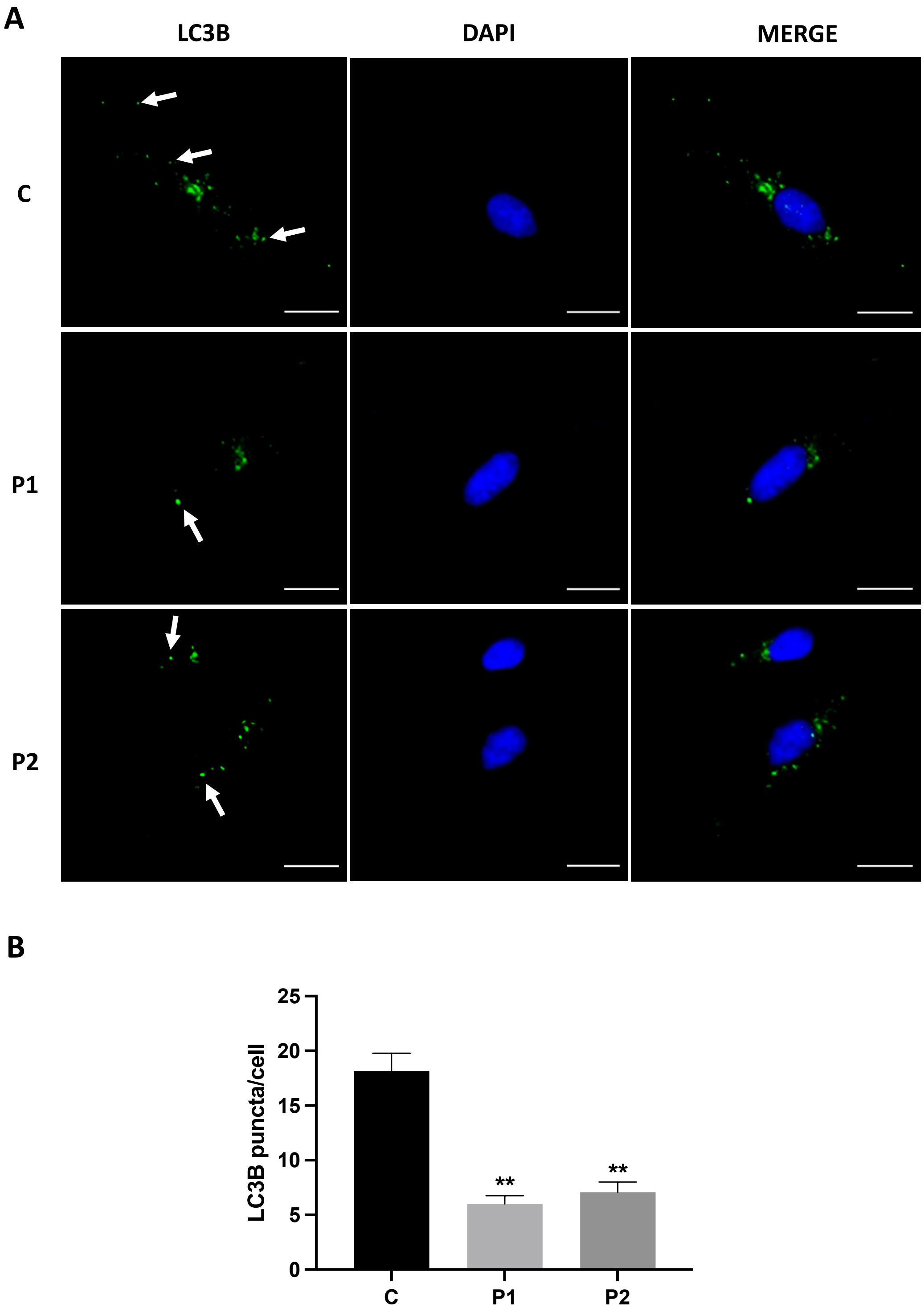

Supplementary Figure S 3. LC3B expression levels by immnunofluorescence microscopy. (**A**) Control (C) and BPAN cells (P1 and P2) were immunostained against LC3B and visualized under widefield fluorescence microscope. Nuclei were revealed by DAPI staining. (**B**) LC3B (white arrows) puncta quantification. Data represent the mean ± SD of three separate experiments (at least 100 cells for each condition and experiment were analysed). **p<0.005 between Control and BPAN fibroblasts. Scale bars=20 µm.

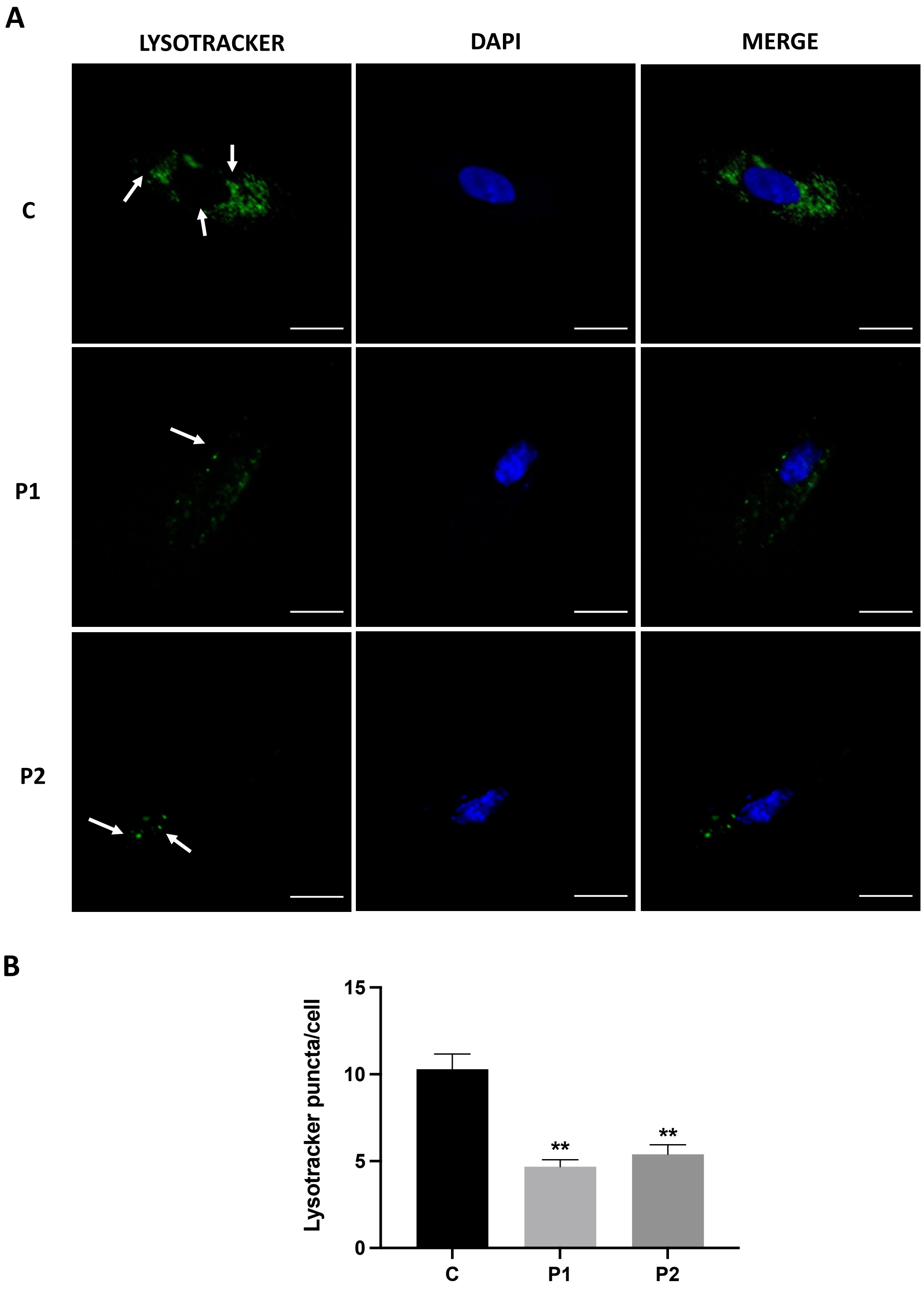

Supplementary Figure S4. Lysosomal compartment in Control and BPAN cells. (**A**) Control (C) and BPAN cells (P1 and P2) were stained with Lysotracker and visualized under widefield fluorescence microscopy. Nuclei were revealed by Hoechst staining. (**B**) Lysotracker puncta (white arrows) quantification. Data represent the mean ± SD of three separate experiments (at least 100 cells for each condition and experiment were analysed). **p<0.005 between Control and BPAN fibroblasts. Scale bars=20 μm.

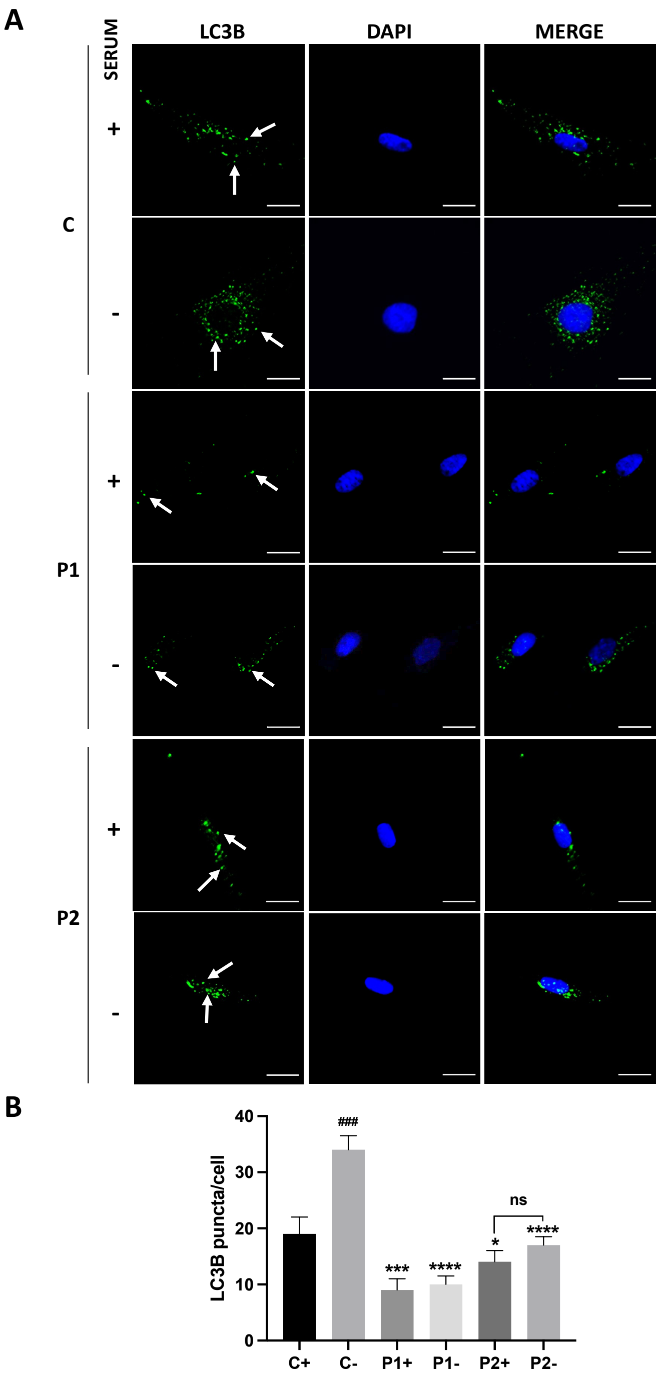

Supplementary Figure S5. Autophagy induction by serum deprivation in Control and BPAN cells. (**A**) Representative image of Control (C) and BPAN cells (P1 and P2) immunostained against LC3B. Autophagy was induced by serum deprivation for 24 hours. (**B**) Quantification of LC3B puncta (white arrows) per cell. Data represent the mean ± SD of four separate experiments (at least 100 cells for each condition and experiment were analysed). *p<0.05, ***p<0.0005 **** p<0.0001; ###p>0.0005 between the presence and the absence of serum. Scale bars=20 µm.

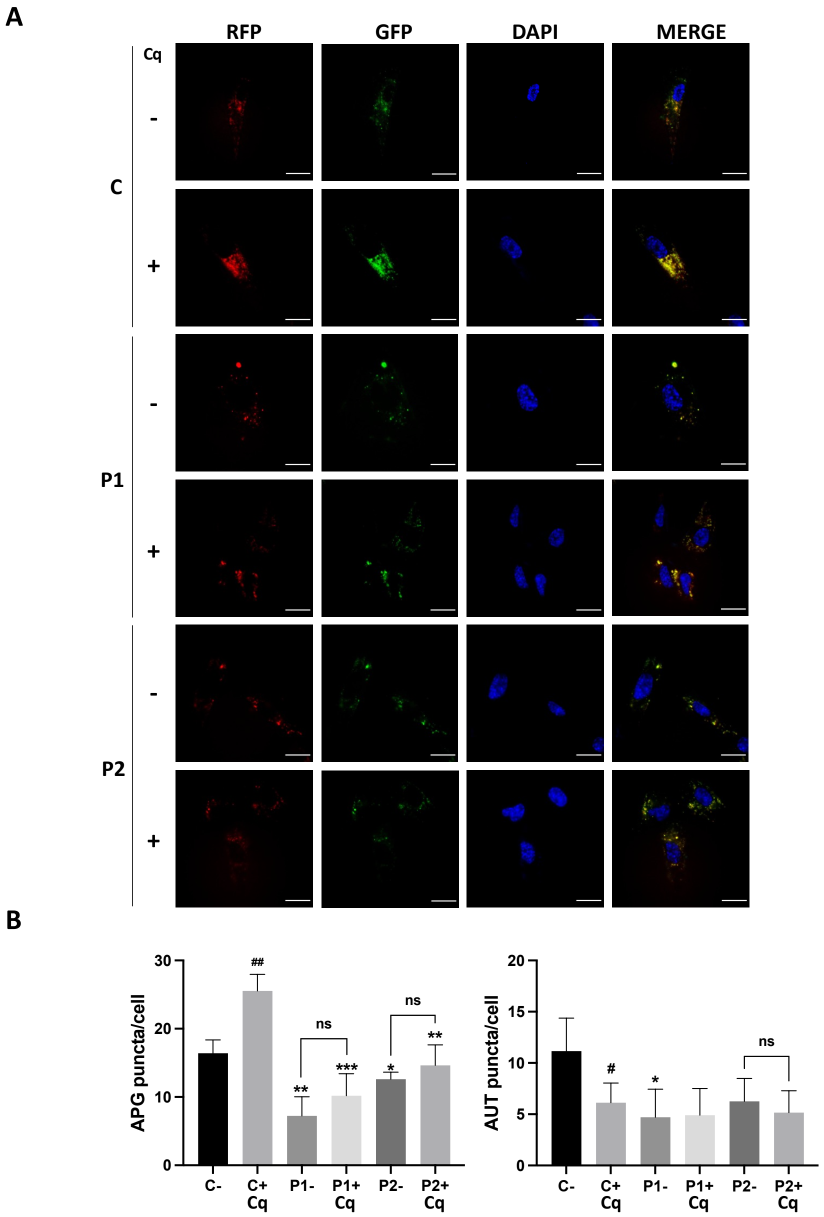

Supplementary Figure S6. Autophagosome formation in Control and BPAN cells. Tandem RFP-GFP-LC3B autophagy sensor was used to determine basal autophagosome formation and after autophagy inhibition by chloroquine (Cq). Cells were then incubated with either vehicle or 90 µM chloroquine (Cq) for 16h and imaged. Scale bars=20 µm. (**A**) Representative fluorescence microscopy images of RFP, GFP and MERGE channels of control and BPAN cells. (**B**) Quantification of RFP and GFP-positive puncta. All images were taken with a widefield fluorescence microscope using a 40X Plan Apo oil objective with standard filter sets for GFP and RFP. Data represent the mean±SD of four separate experiments. *p<0.05, **p<0.005 between BPAN cells and controls; $^{\#}$p<0,05 between the presence and the absence of Cq. APG=autophagosomes; AUT=autolysosomes.

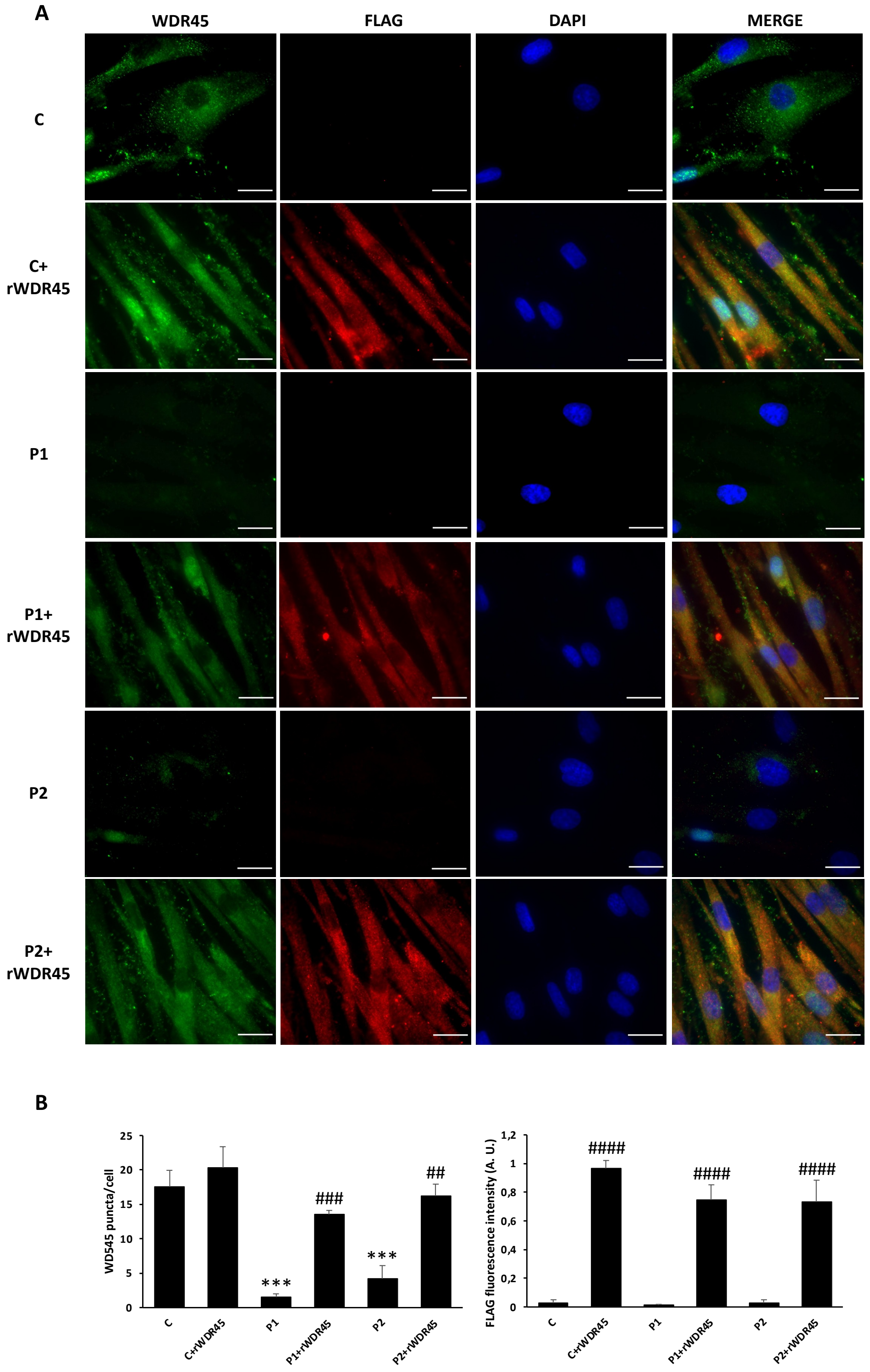

Supplementary Figure S7. (A) Representative immunofluorescence images of WDR45 and FLAG in control and mutant fibroblasts P1 and P2 expressing recombinant WDR45 (rWDR45). Nuclei were revealed by DAPI staining. (B) Quantification of WDR45 and FLAG signals. Data represent the mean ± SD (at least 100 cells for each condition and experiment were analysed). $^{***}p<0.005$ between Control and BPAN fibroblast; $^{\#\#\#\#}p<0,0001$ between the presence and the absence of rWDR45. Scale bars=20 µm.

Supplementary Figures

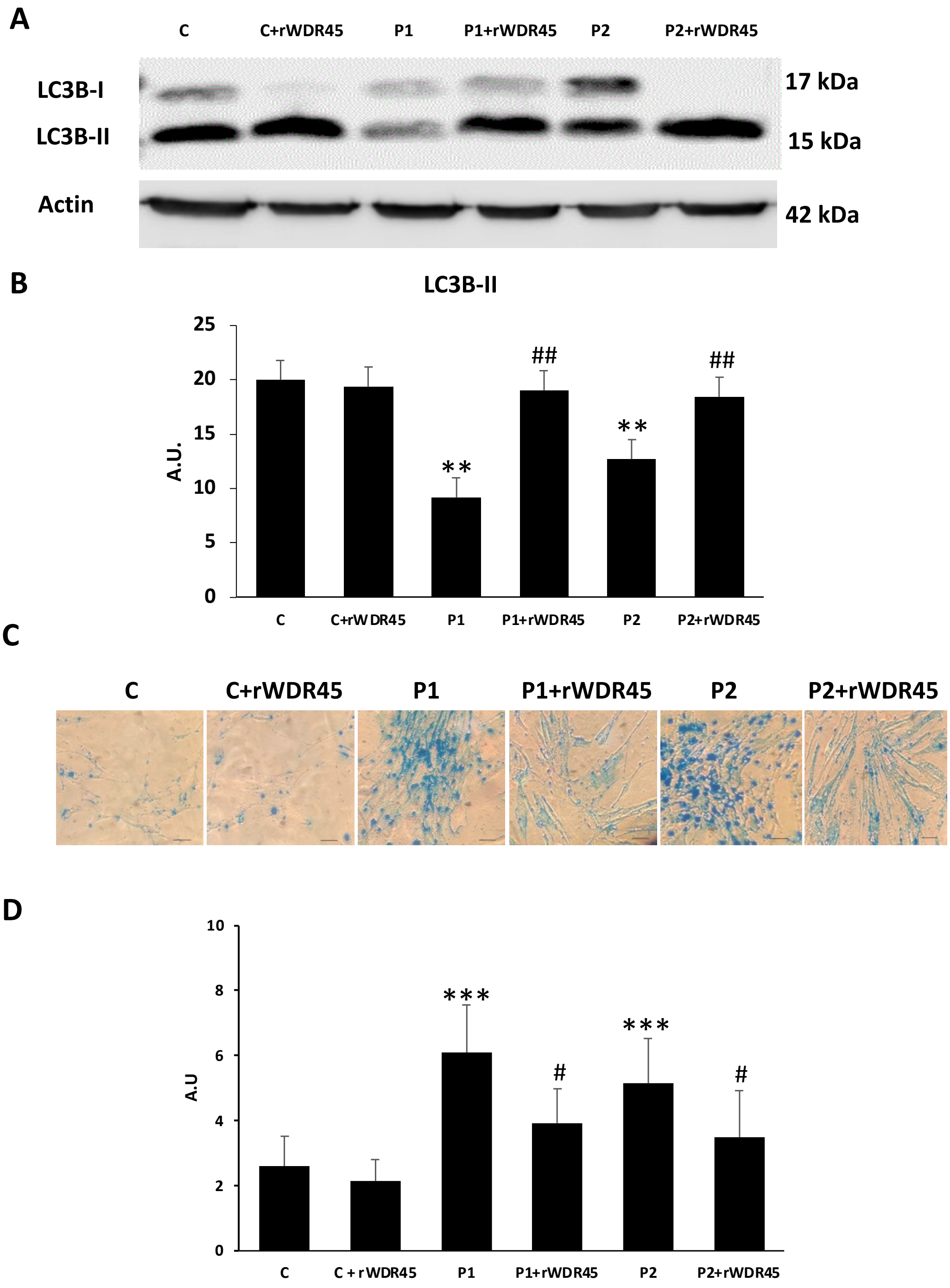

Supplementary Figure S8. (**A**) Representative immunoblot of LC3B levels in total cell lysates isolated from control fibroblasts and mutant fibroblasts P1 and P2 expressing recombinant WDR45 (rWDR45). Equal loading was verified by immunoblotting with actin antibody. (**B**) LC3B-II expression levels quantification by were analyzed by the Image J software. (**C**) Prussian Blue staining of control cells (C) and BPAN fibroblasts (P1 and P2) expressing recombinant WDR45 (rWDR45). (**D**) Quantification of Prussian Blue staining Images were analyzed by the Image J software. Data represent the mean ± SD (at least 100 cells for each condition and experiment were analysed). **p<0.005, ***p<0.0005 between Control and BPAN fibroblast; $^{\#}$p<0,05, $^{\#\#}$p<0,005 between the presence and the absence of rWDR45. Scale bars=20 µm.

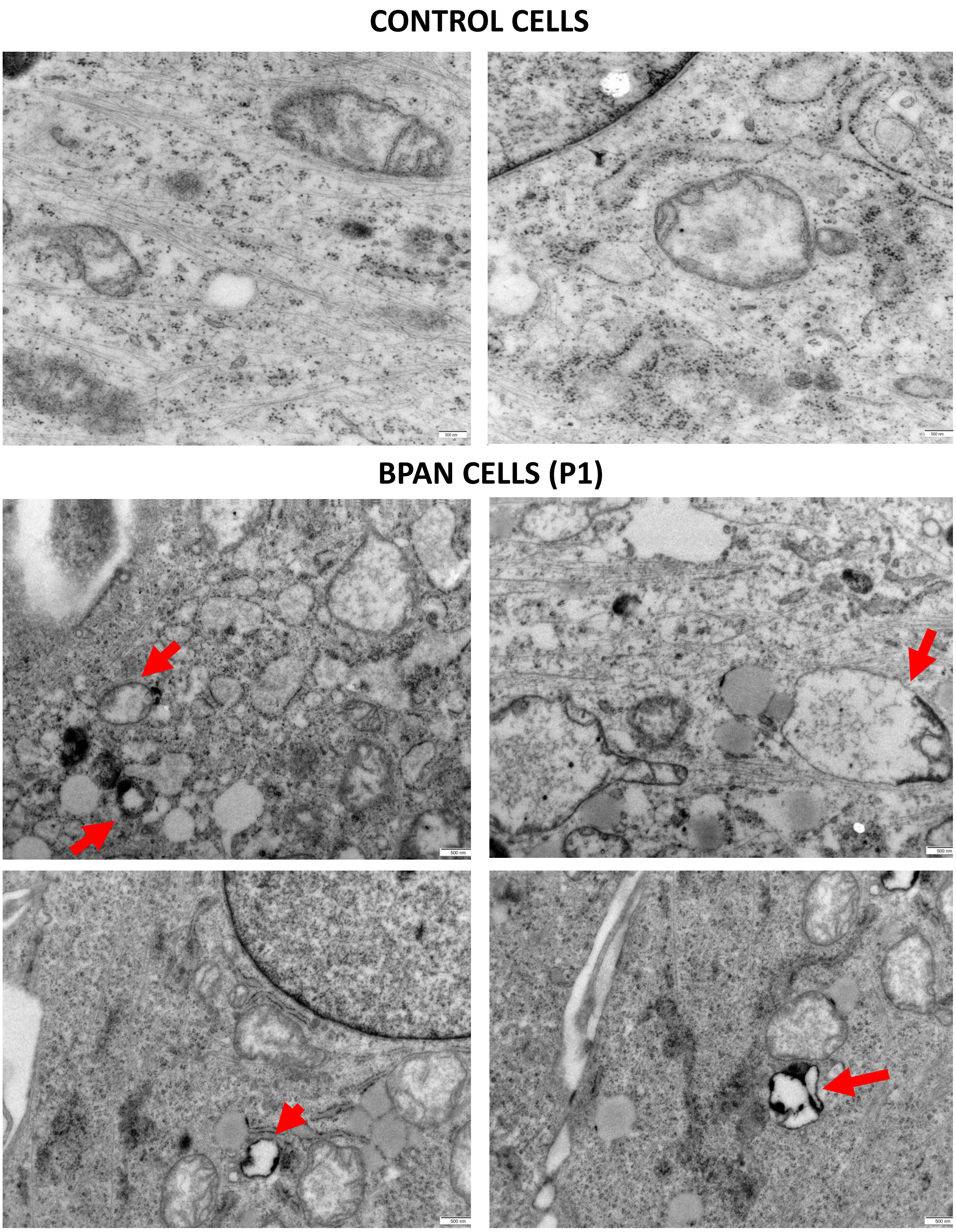

Supplementary Figure S9. Electron microscopy examination of Control and BPAN cells. Representative TEM images of Control and BPAN fibroblasts (P1). Control cells showed normal mitochondrial morphology. BPAN cells showed mitochondrial vacuolization, and condensation/lateralization of mitochondrial membranes (red arrows). Scale bars=500 nm.

BPAN CELLS (P1)

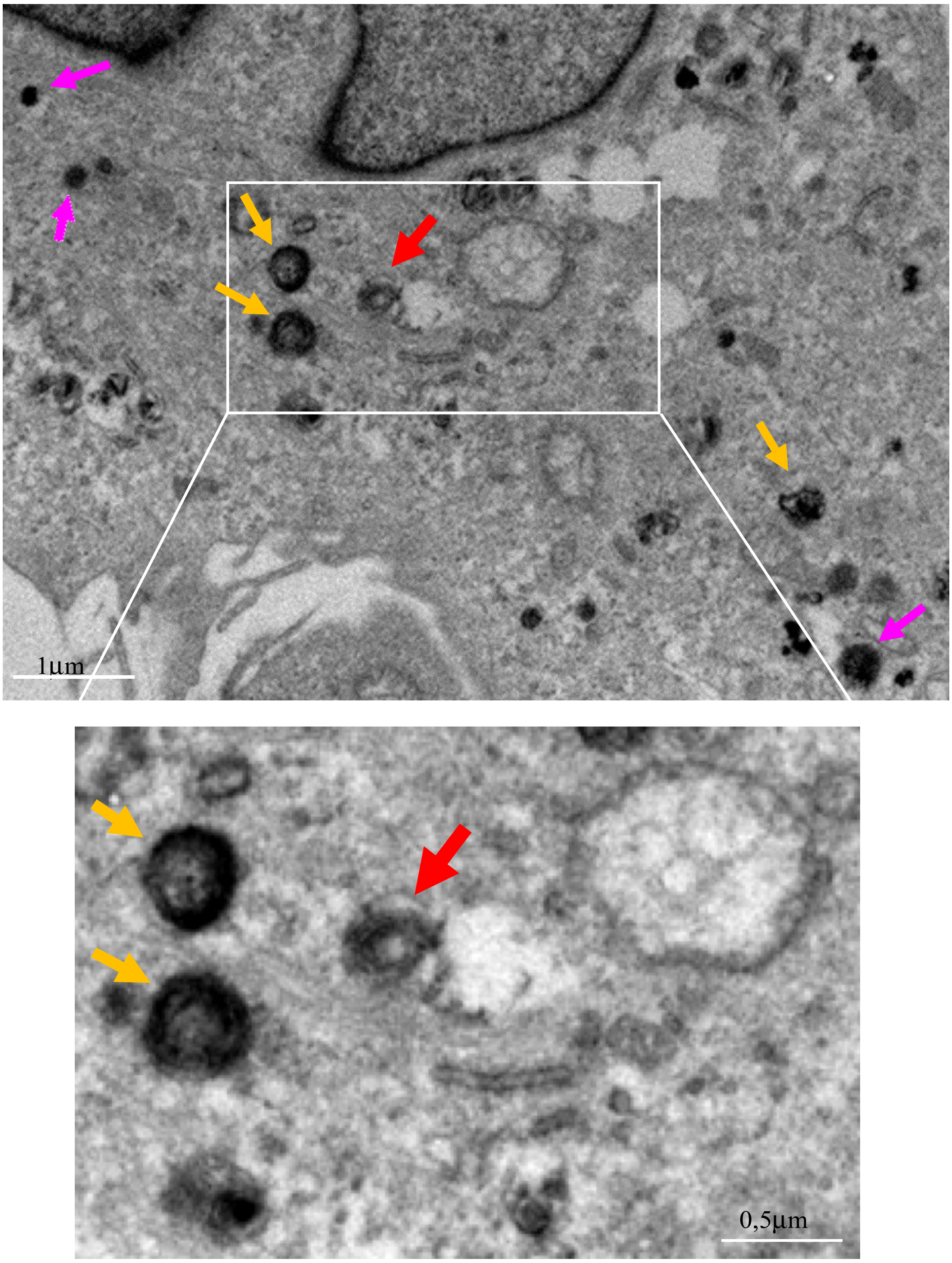

Supplementary Figure S10. Electron microscopy examination of BPAN cells. Representative images of BPAN fibroblasts (P1). BPAN cells showed mitochondrial vacuolization, and condensation/lateralization of mitochondrial membranes (red arrow). BPAN cells showed the accumulation of lipofuscin-like aggregates (orange arrows) and lipofuscin granules (pink arrows). Top panel, scale bar=1µm. Bottom panel, magnification of an area (white rectangle) of the figure in the Top panel, scale bar=0,5 µm.

BPAN CELLS (P2)

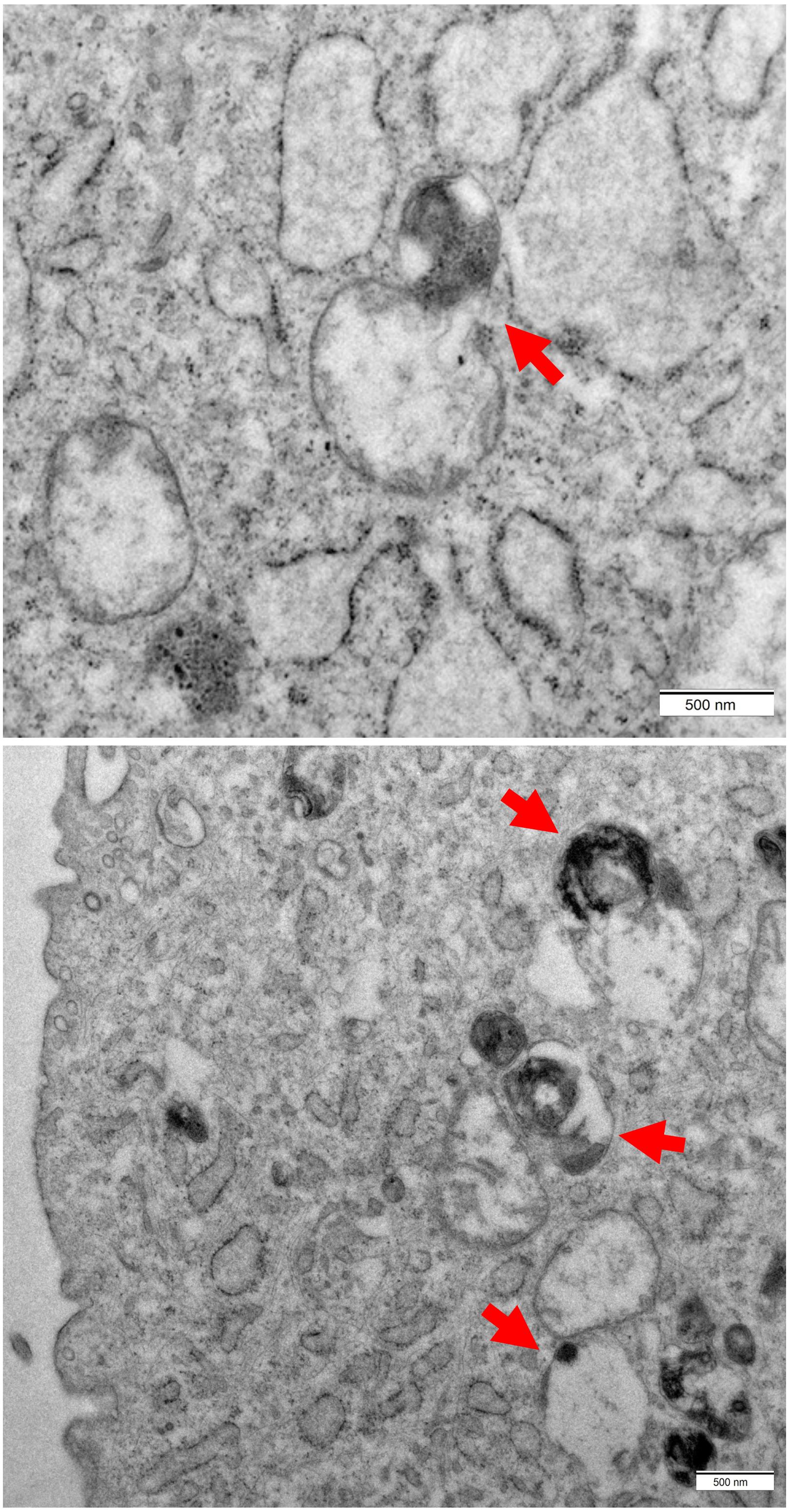

Supplementary Figure S 11. Electron microscopy examination of mitochondria alterations in BPAN cells. Representative images of BPAN fibroblasts (P2). BPAN cells showed mitochondrial vacuolization, and condensation/lateralization of mitochondrial membranes (red arrows). Scale bars=500 nm.

BPAN CELLS (P1)

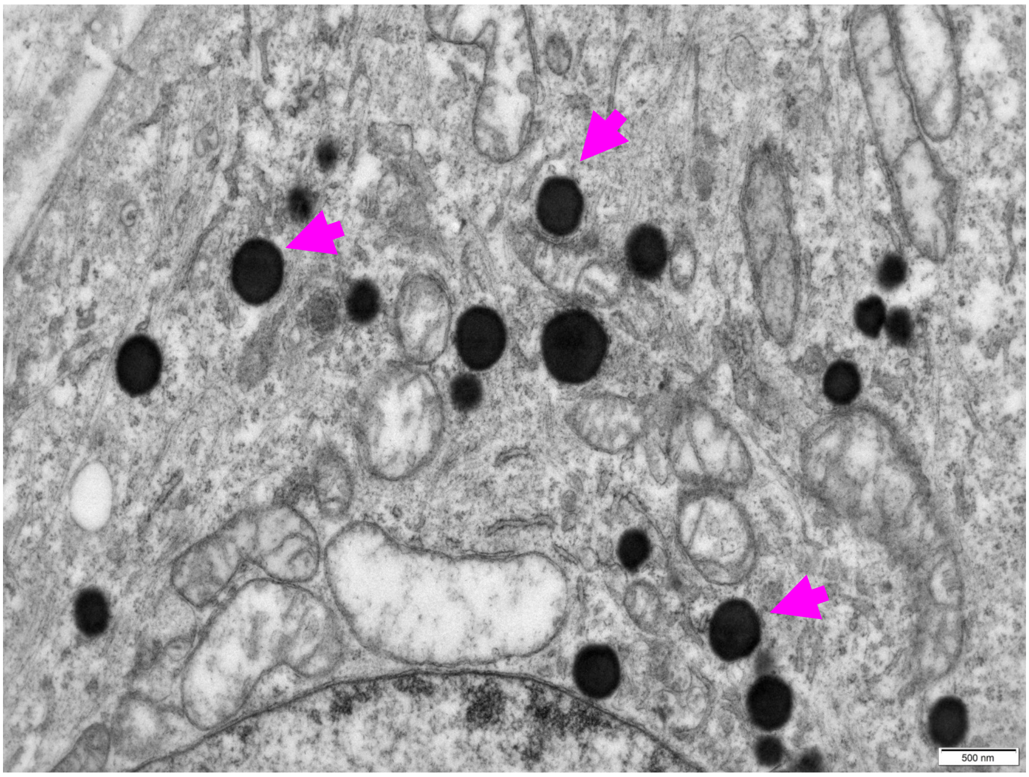

BPAN CELLS (P2)

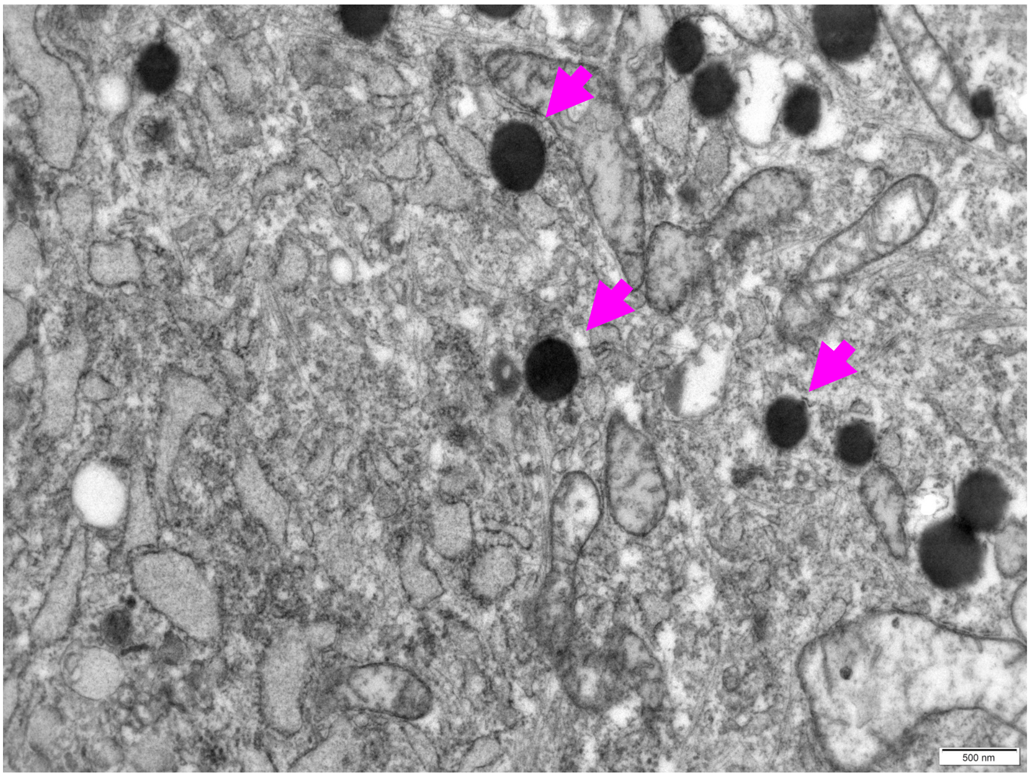

Supplementary Figure S12. Electron microscopy examination of lipofuscin granules in BPAN cells. Representative images of BPAN fibroblasts (P1 and P2). BPAN cells showed typical lipofuscin granules (pink arrows). Scale bars=500 nm.

CONTROL CELLS

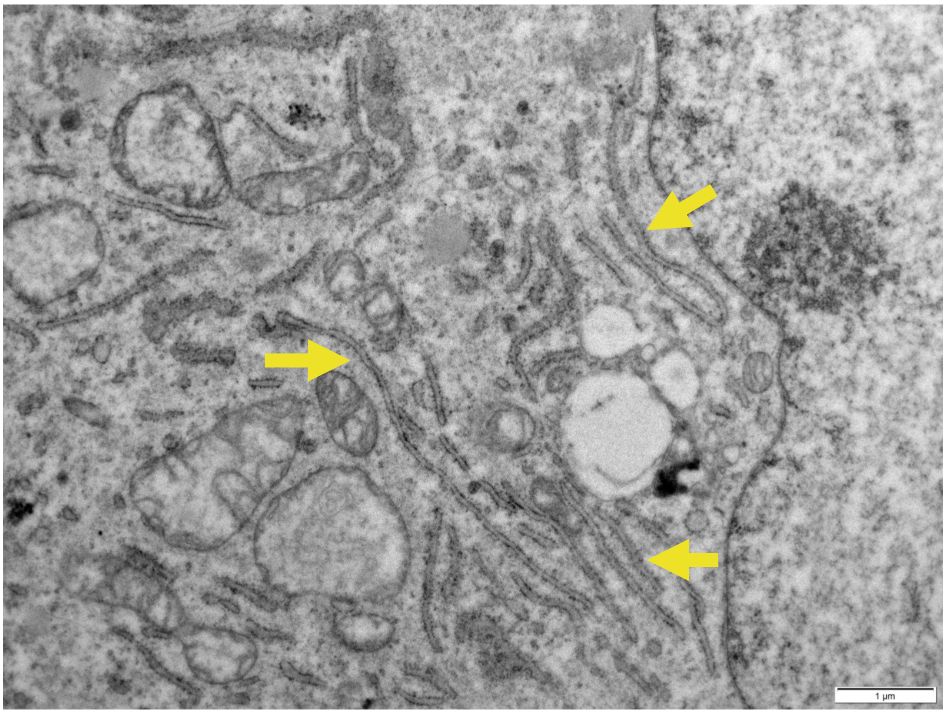

BPAN CELLS (P2)

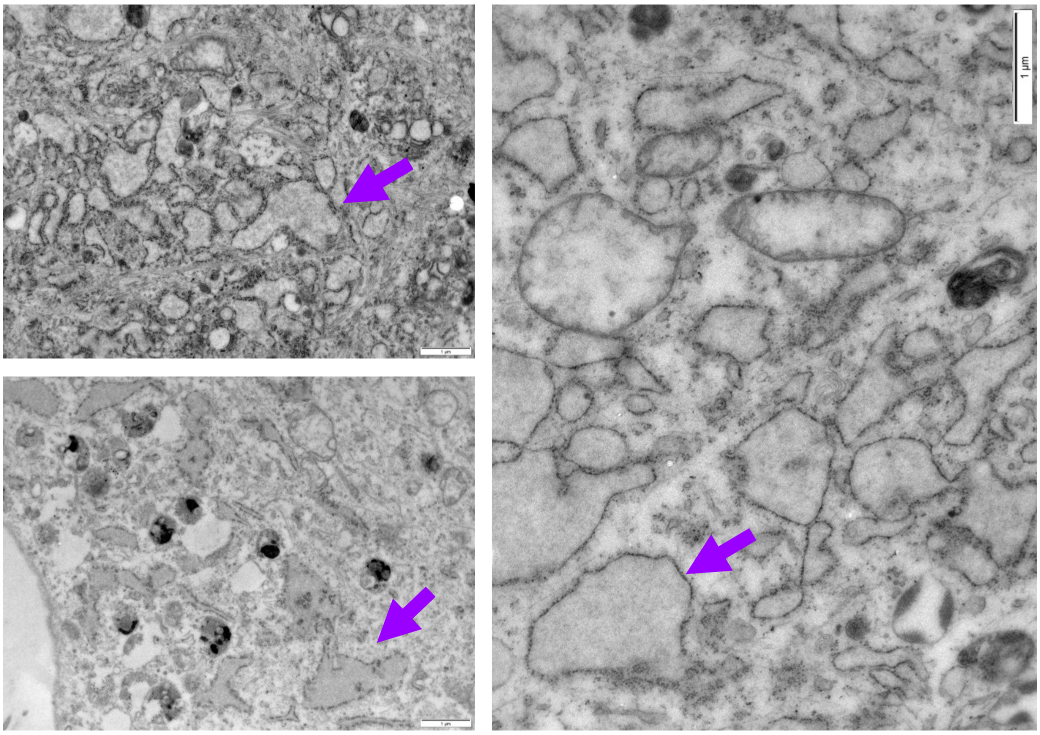

Supplementary Figure S13. Electron microscopy examination of RER in Control and BPAN cells. Representative images of Control (C) and BPAN fibroblasts (P2). Control cells showed normal RER size (yellow arrows). BPAN cells showed RER dilation (purple arrows). Scale bars= 1 µm.

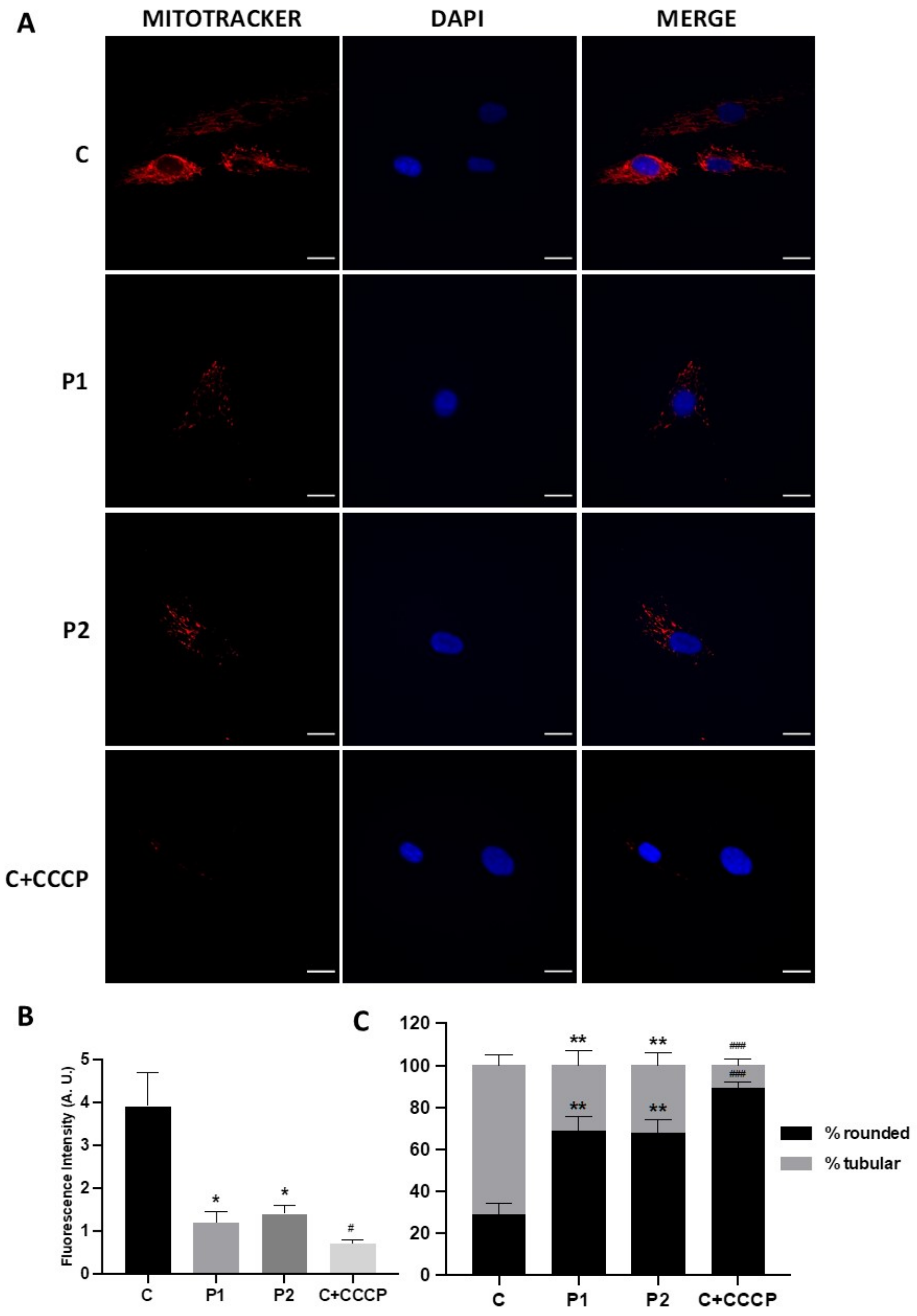

Supplementary Figure S14. Mitochondrial polarization and network in Control and BPAN cells. (**A**) Representative images of control (C) and BPAN fibroblasts (P1 and P2) stained with MitoTracker^{TM} Red CMXRos. As a positive control of membrane depolarization, we used 100 µM CCCP for 4 h in control cells. Scale bar = 20 µm. (**B**) Fluorescence quantification of MitoTracker signal. (**C**) Quantification of tubular and rounded percentage of mitochondria in control and BPAN fibroblasts. Data represent the mean ± SD of three separate experiments (at least 100 cells for each condition and experiment were analysed). *$p<0.05$ between BPAN cells and controls; $^{\#}p<0.05$ between the presence and the absence of CCCP. A. U.: arbitrary units.

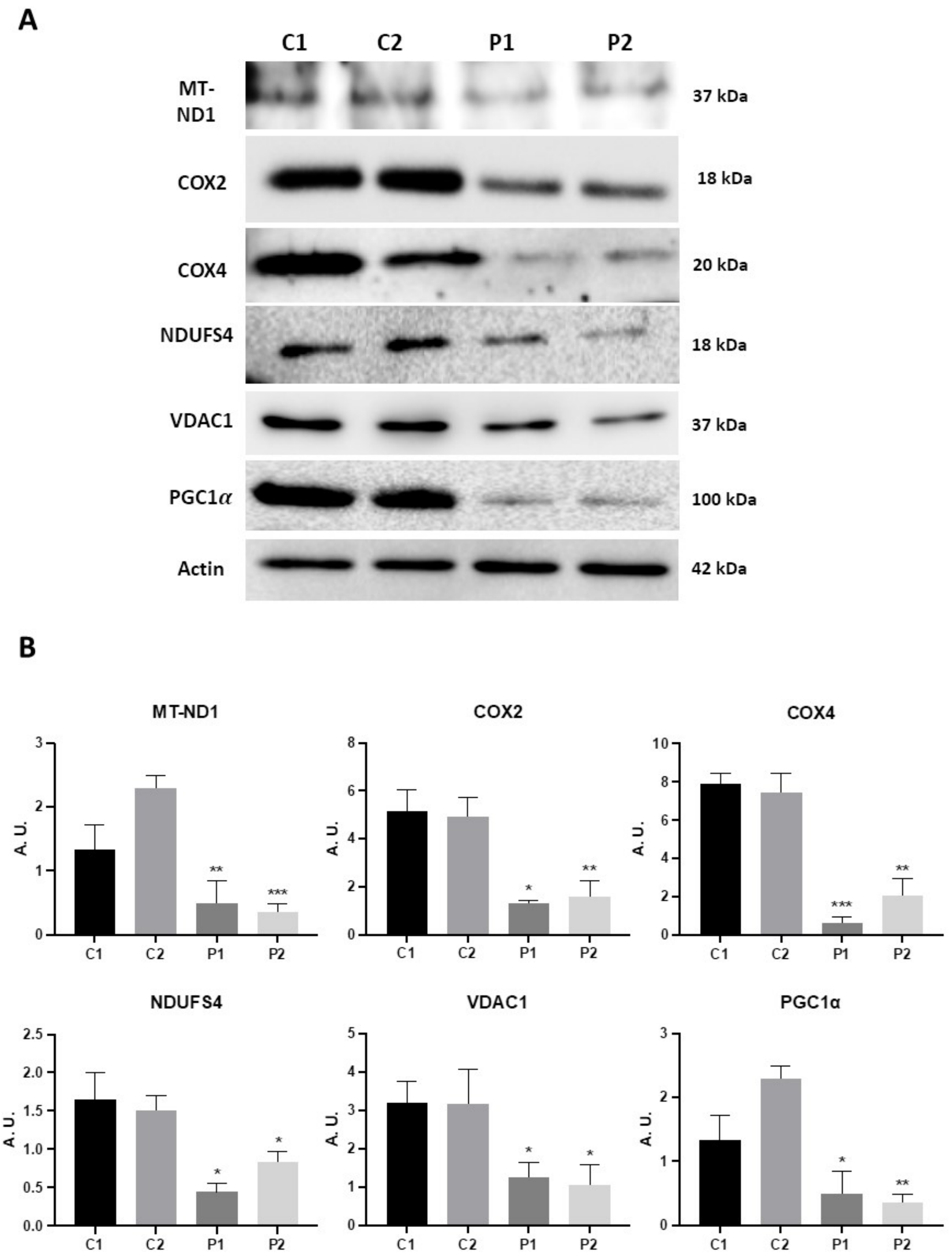

Supplementary Figure S15. Mitochondrial protein expression levels in Control and BPAN cells. (**A**) Immunoblotting analysis of cellular extracts from controls (C1 and C2) and BPAN patient cell lines P1 and P2. Protein extracts (50 µg) were separated on a SDS polyacrylamide gel and immunostained with antibodies against MTND1, COX2, COX4, NDUFS4, VDAC1 and PGC1α. Actin was used as a loading control. (**B**) Densitometry of the Western blotting. For controls cells (C1 and C2), data are the mean±SD of the two control cell lines. Data represent the mean±SD of three separate experiments. *p<0.05, **p<0.005, ***p<0.0005 between BPAN cells and controls. A.U., arbitrary units.

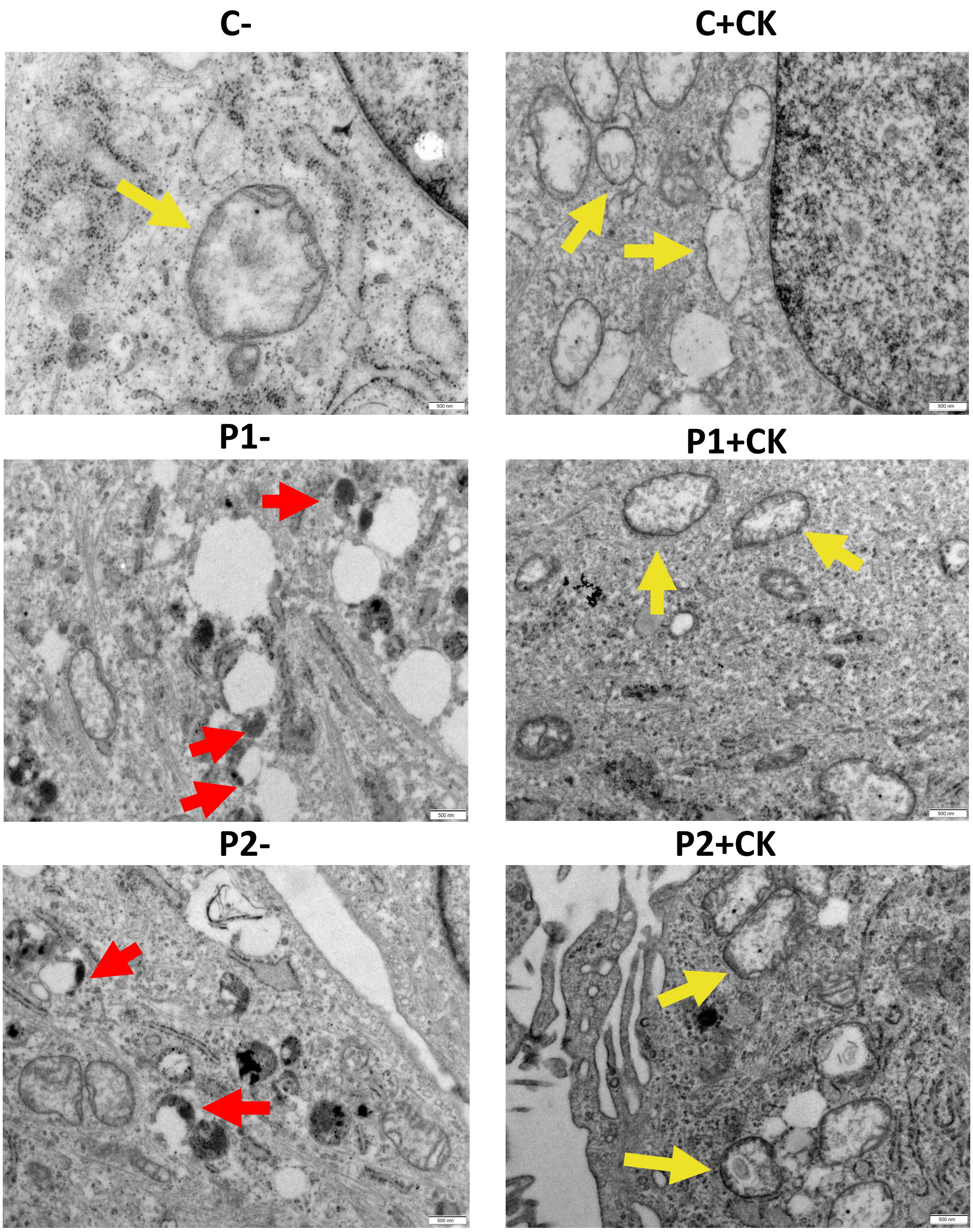

Supplementary Figure S16. Effect of antioxidants on mitochondrial ultrastructure in Control and BPAN cells. BPAN fibroblasts were treated with antioxidants cocktail (CK, 1 μM α-lipoic acid, 10 μM vitamin E and 5 μM pantothenate) for 7 days. Representative TEM images of untreated (-) and treated (+) Control and BPAN fibroblasts (P1 and P2). Control cells displayed normal mitochondria (yellow arrows). BPAN cells showed mitochondrial vacuolization and lateralization/condensation of mitochondrial membranes (red arrows) which was markedly reduced after antioxidant treatment (P1+ and P2+) (yellow arrows). Scale bars=500 nm.

RER DILATATION

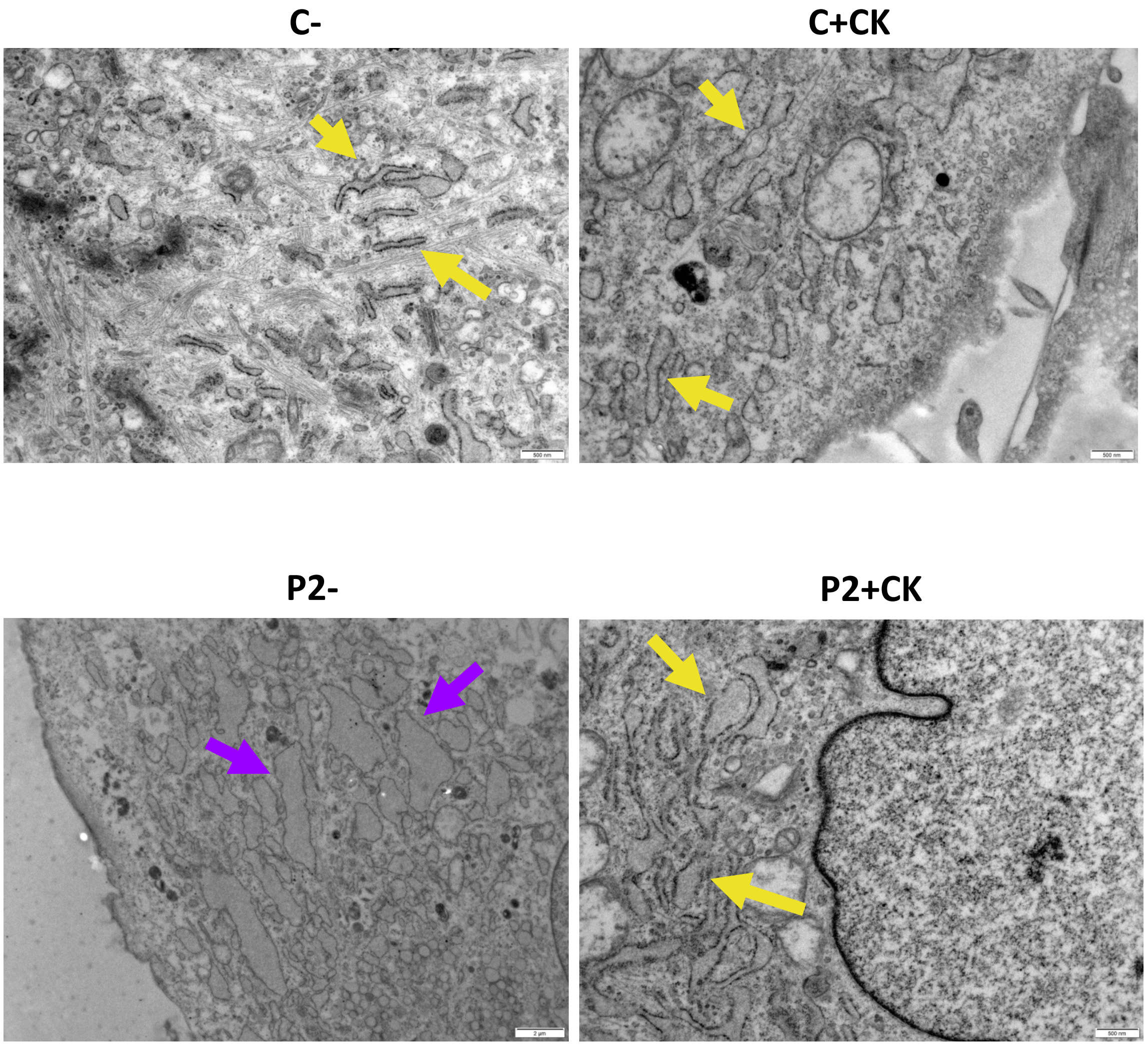

Supplementary Figure S17. Effect of antioxidants on RER dilatation in BPAN cells. BPAN fibroblasts were treated with antioxidants cocktail (CK, 1 µM α-lipoic acid, 10 µM vitamin E and 5 µM pantothenate) for 7 days. Representative TEM images of untreated (-) and treated (+) BPAN fibroblasts (P2- and P2+). Control cells displayed normal RER (yellow arrows). BPAN cells showed RER dilation (purple arrows) which was markedly reduced after antioxidant treatment (yellow arrows). Scale bars= 500 nm.

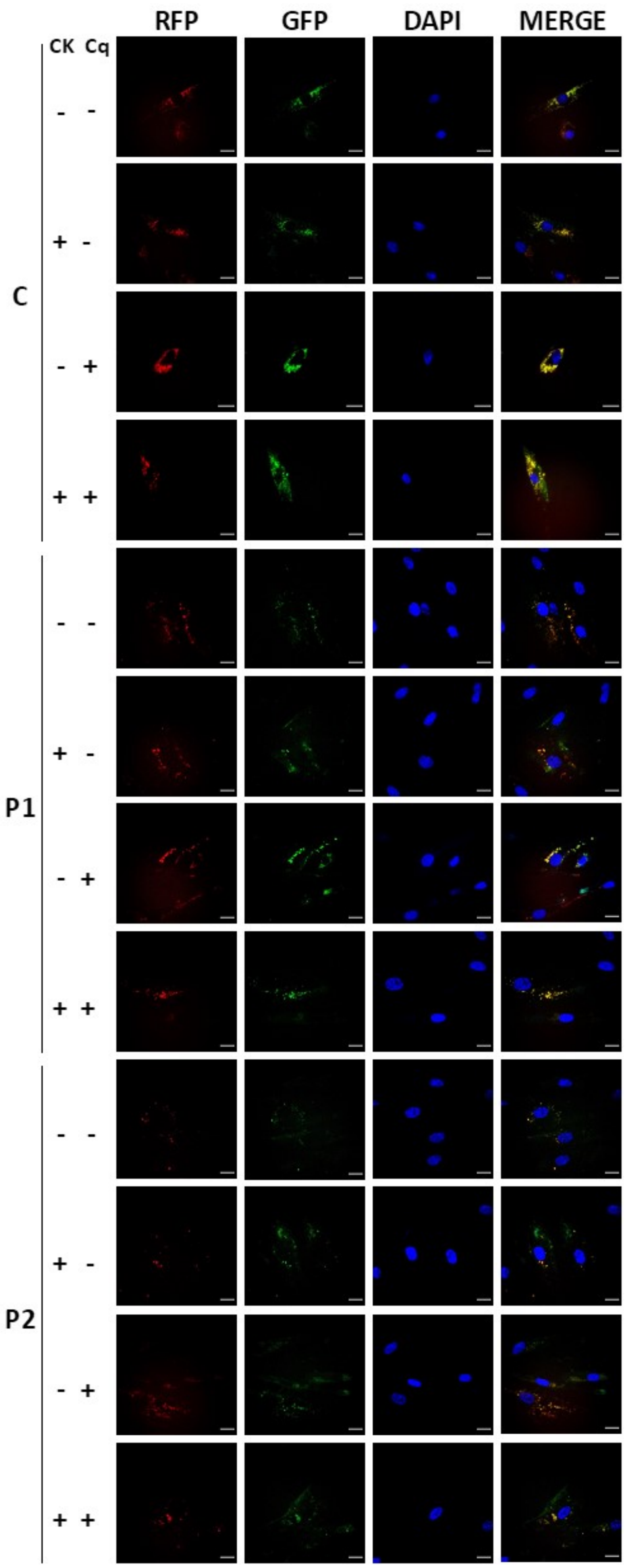

Supplementary Figure S18. RFP, GFP, DAPI and merge channels corresponding to the Tandem Sensor RFP-GFP-LC3B assay of Figure 14. Control (C) and BPAN (P1 and P2) untreated (-) and treated (+) with antioxidant cocktail (CK, 1 μM α-lipoic acid, 10 μM vitamin E and 5 μM pantothenate) and 90 μM chloroquine (Cq). Scale bars=20 μm.

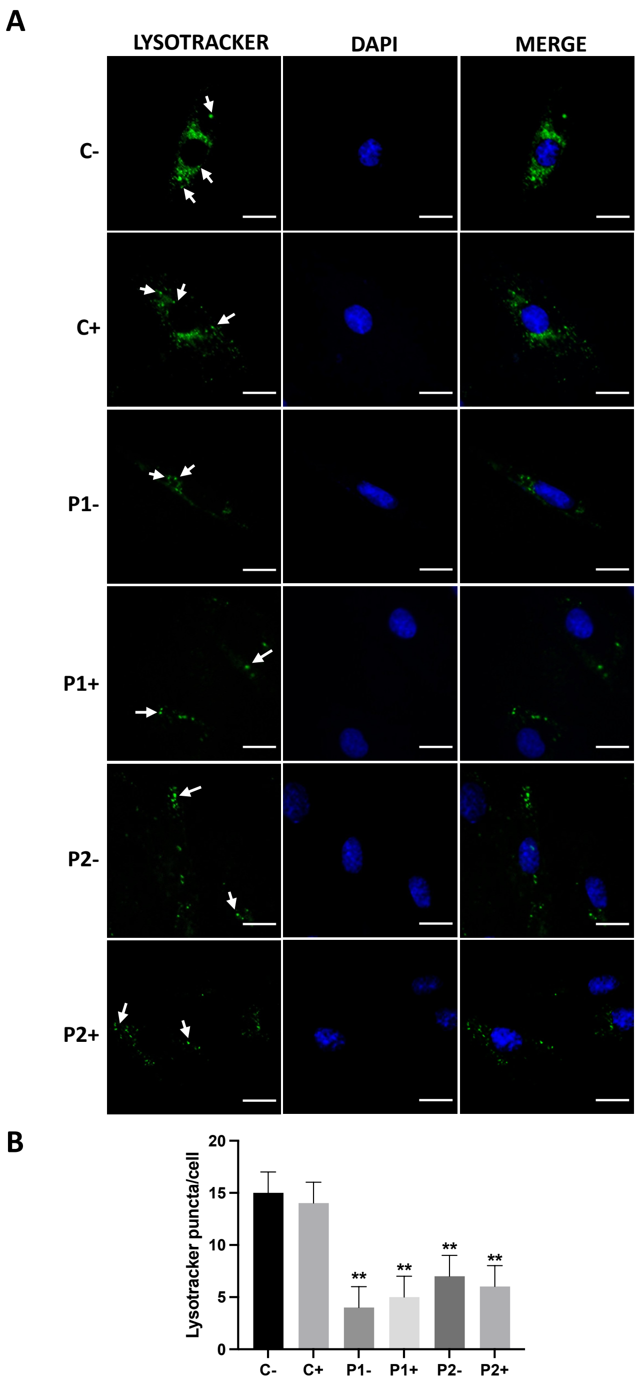

Supplementary Figure S 19. Effect of antioxidant treatment on lysosomal compartment. Control (C) and BPAN fibroblasts were treated with antioxidants (1 μM α-lipoic acid, 10 μM vitamin E and 5 μM pantothenate) for 7 days. Next, cells were stained with Lysotracker. Nuclei were revealed by Hoechst staining. Scale bars=20 μm.(**A**) Representative fluorescence images of untreated (-) and treated (+) Control and BPAN cells. (**B**) Lysotracker puncta (white arrows) quantification. Data represent the mean ± SD of three separate experiments (at least 100 cells for each condition and experiment were analysed). **p<0.005 between Control and BPAN fibroblasts.

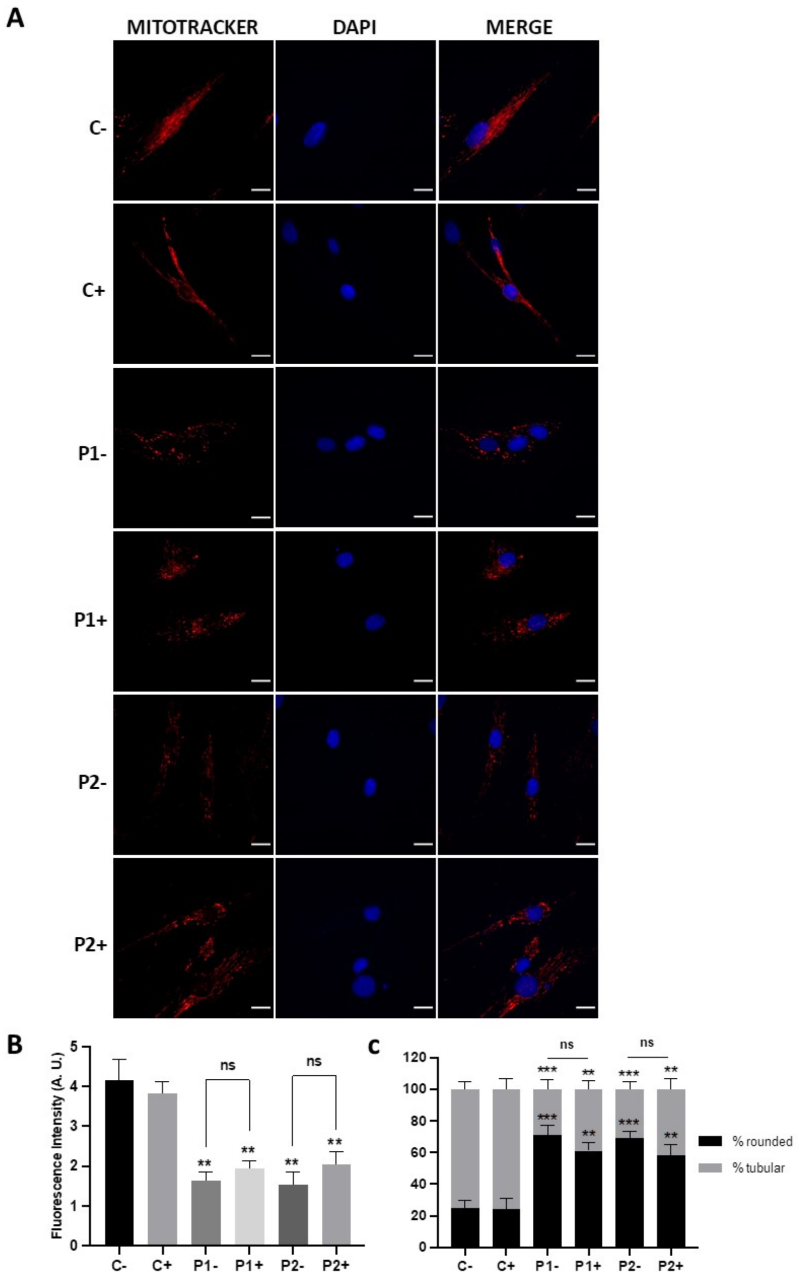

Supplementary Figure S 20. Effect of antioxidant treatment on Mitochondrial polarization and mitochondrial network. Control (C) and BPAN fibroblasts were treated with antioxidants (1 µM α-lipoic acid, 10 µM vitamin E and 5 µM pantothenate) for 7 days. Next, cells were stained with MitoTracker^{TM} Red CMXRos. Scale bars=20 µm. (**A**) Representative images of untreated (-) and treated (+) of Control and BPAN fibroblasts (P1 and P2). (**B**) Quantification of fluorescence intensity. (**C**) Quantification of tubular and rounded mitochondria. Rounded mitochondria were defined as 0.2-0.5 µm and tubular mitochondria as > 0.5 µm. Data represent the mean ± SD of three separate experiments (at least 100 cells for each condition and experiment were analysed). **p<0.005, ***p<0.0005 between Control and BPAN fibroblasts.
