# Supplementary material for: Antioxidants Prevent Iron Accumulation and Lipid Peroxidation, but Do Not Correct Autophagy Dysfunction or Mitochondrial Bioenergetics in Cellular Models of BPAN
Source: Int J Mol Sci. 2023 Sep 26;24(19):14576. doi: 10.3390/ijms241914576 (PMC11340724; doi:10.3390/ijms241914576)
Supplement: Supplementary file 1 [file ijms-24-14576-s001.zip › Uncropped membranes.pdf]

Uncropped gels of figure 1

ULK1

100 kDa

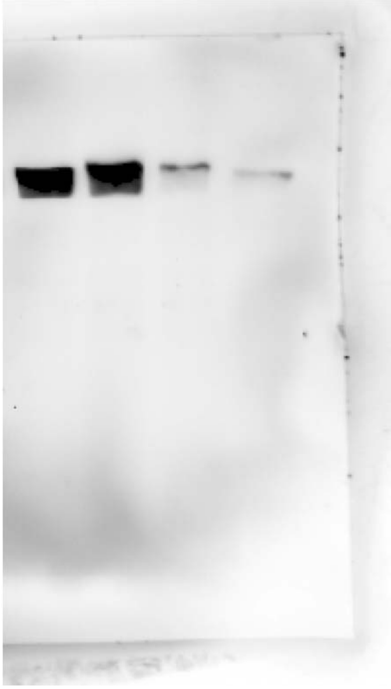

BECN1

60 kDa

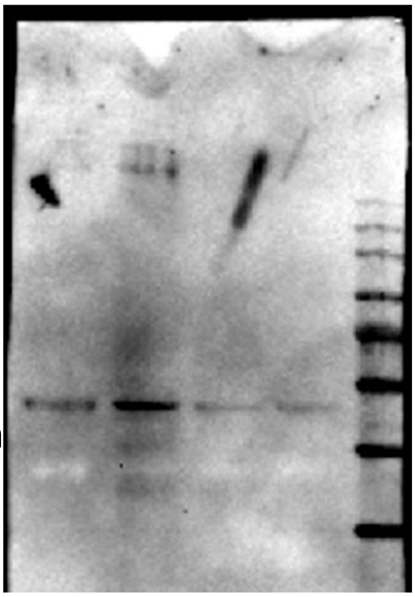

WDR45

45 kDa

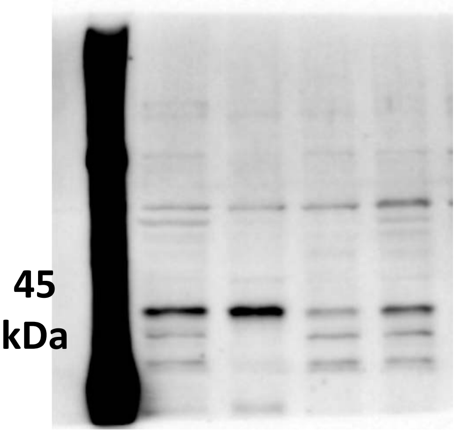

ATG12-ATG5

55 kDa

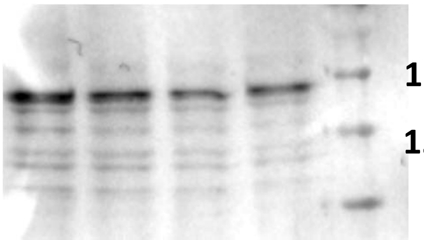

LC3B

17 kDa

15 kDa

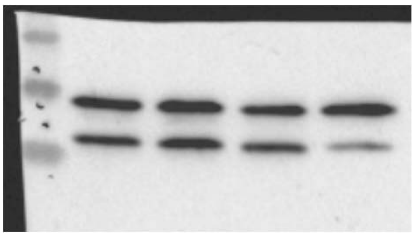

TFEB

62 kDa

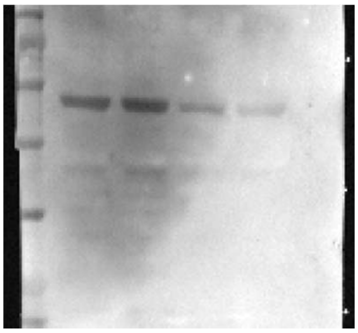

P62

62 kDa

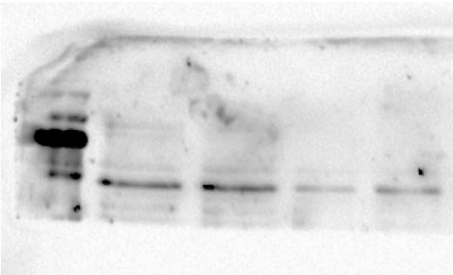

42 kDa

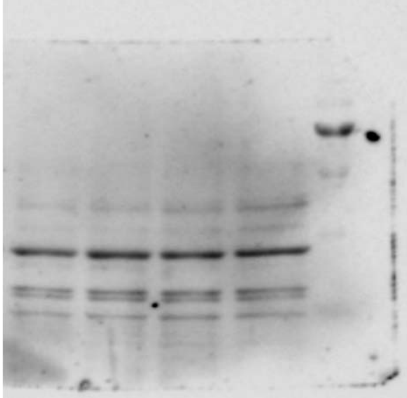

Actin

Uncropped gels of figure 2

LAMP1

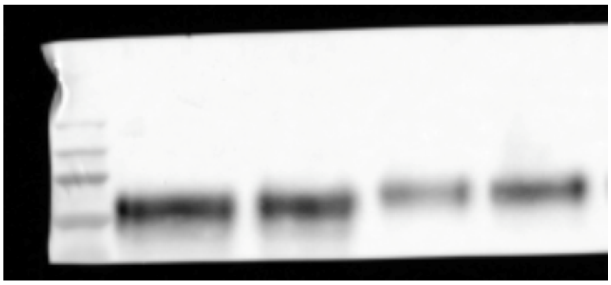

125 kDa

LAMP2

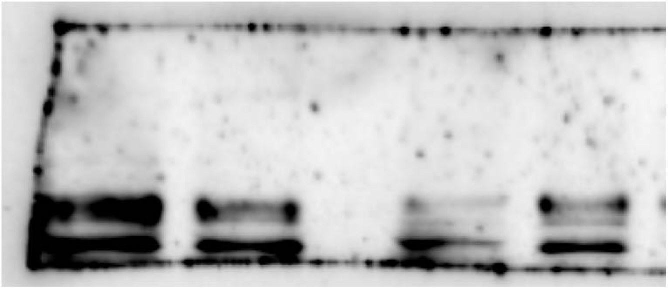

115 kDa

LAMP2-COLORIMETRIC

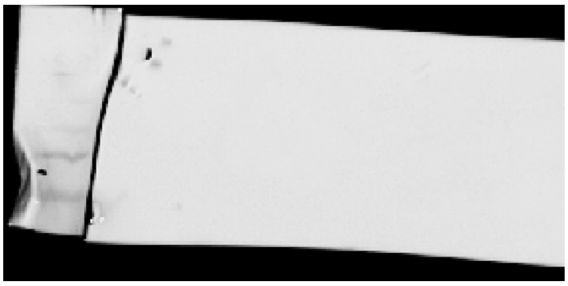

GBA1

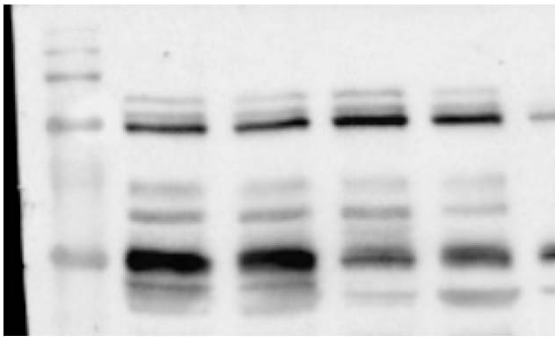

62 kDa

CATC

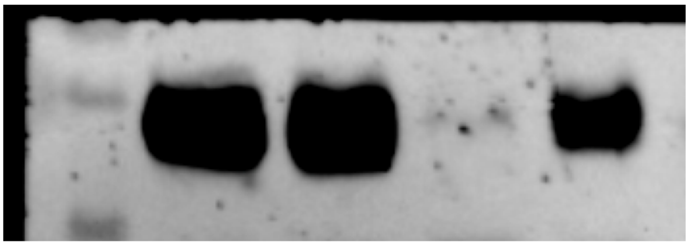

20 kDa

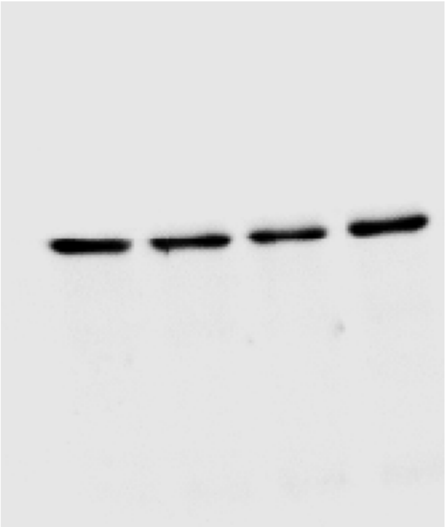

42 kDa

Actin

Uncropped gels of figure 3

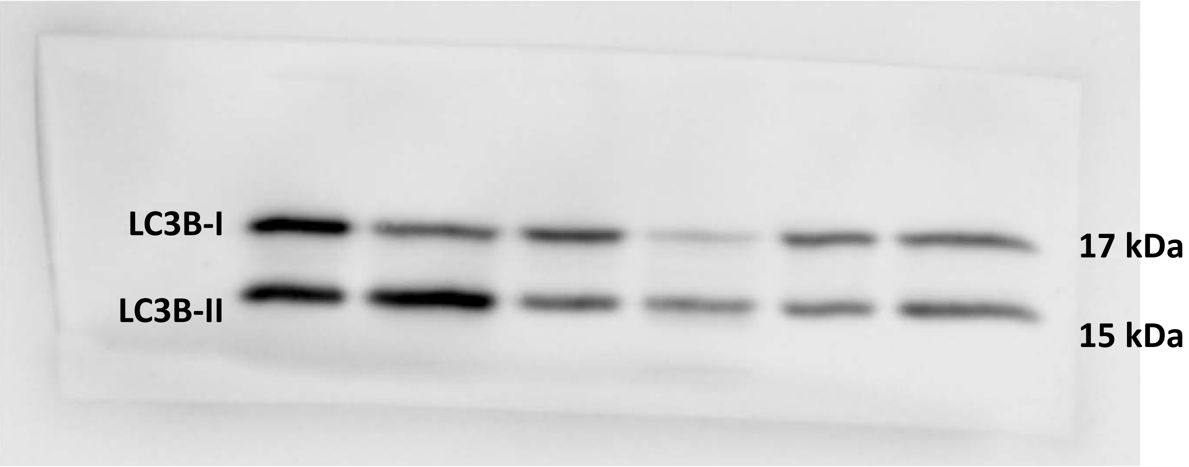

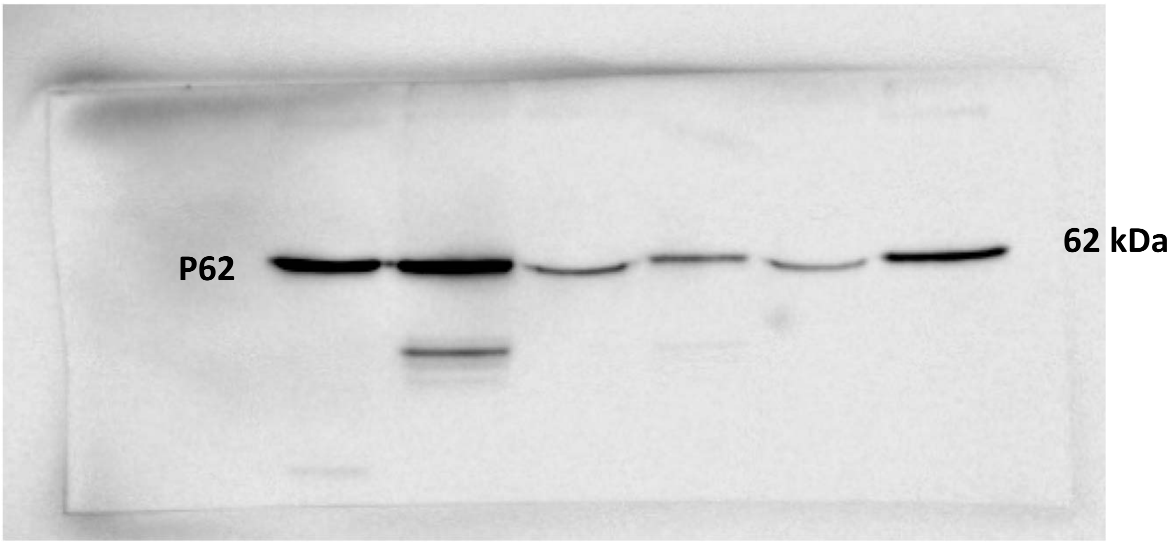

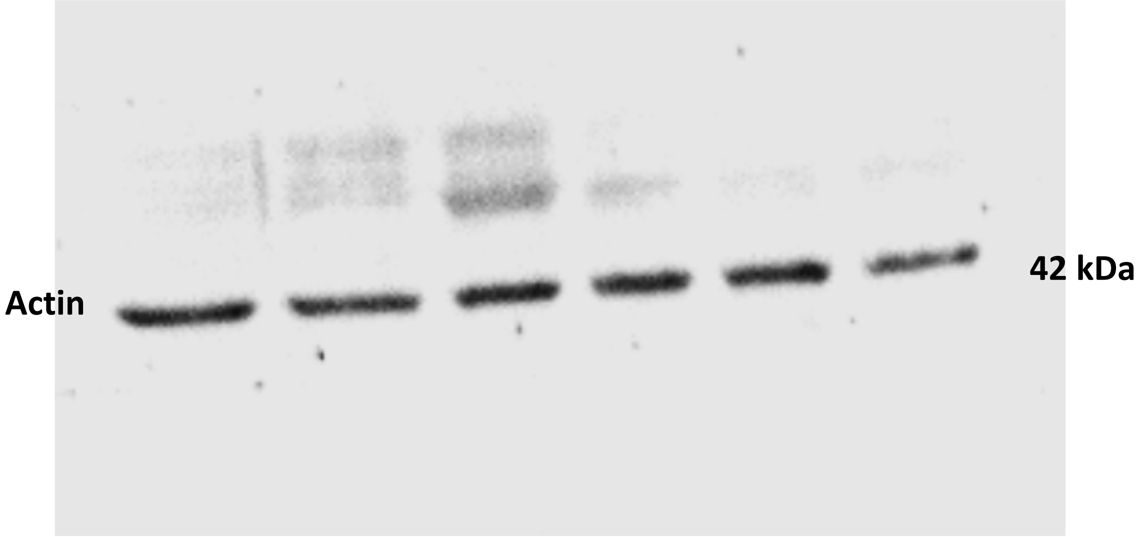

Uncropped gels of figure 7

SLC40A1

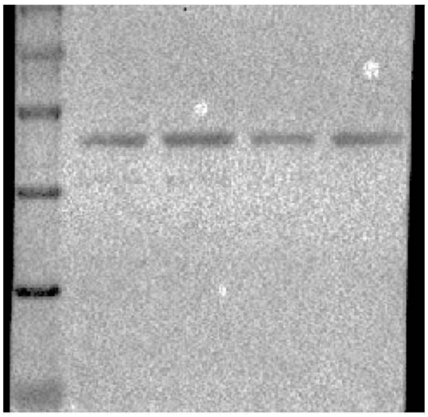

TFR1

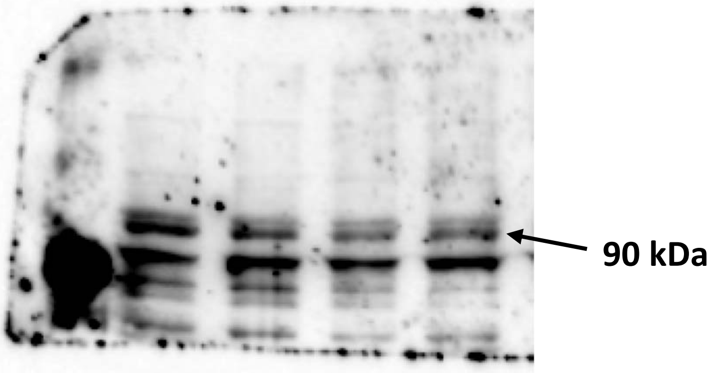

DMT1

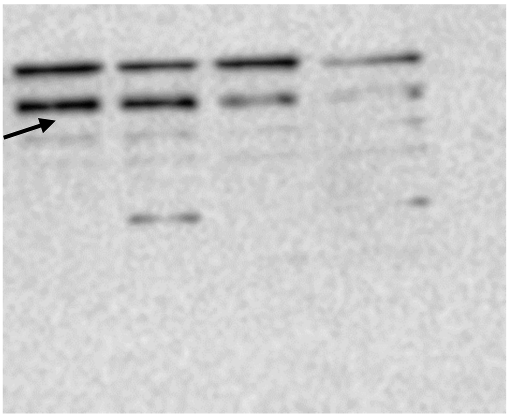

FT

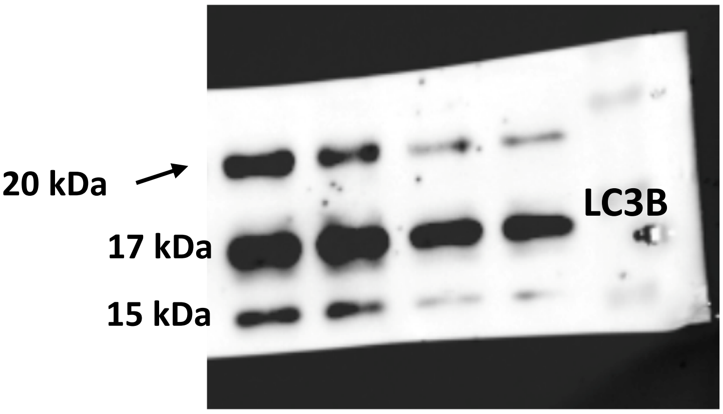

MFRN2

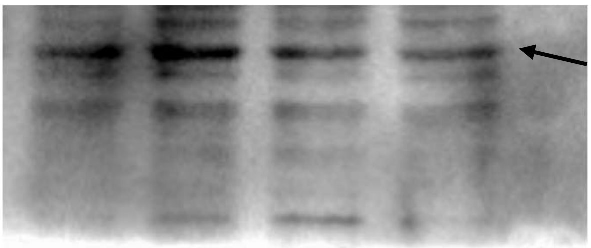

FTMT

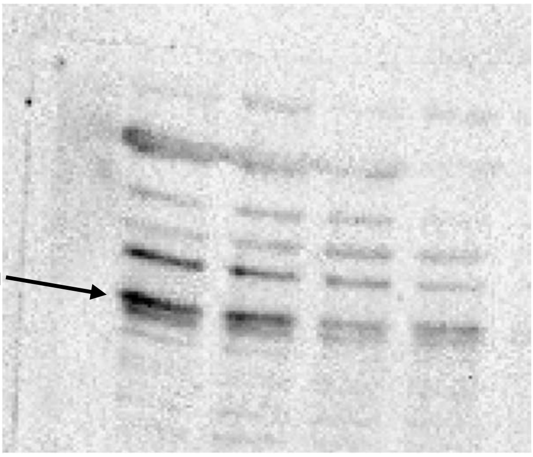

NCOA4

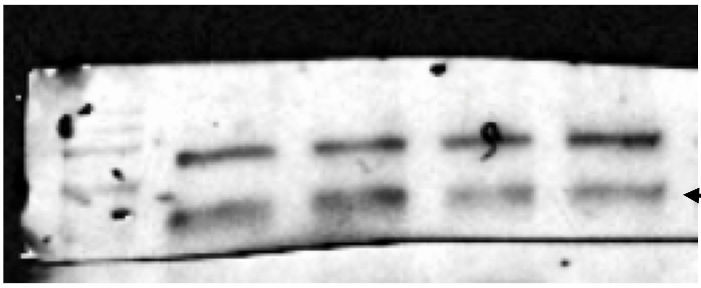

Uncropped gels of figure 7 (continued)

FXN

14 kDa

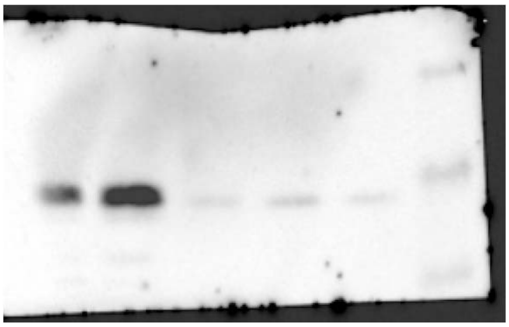

NFS1

50 kDa

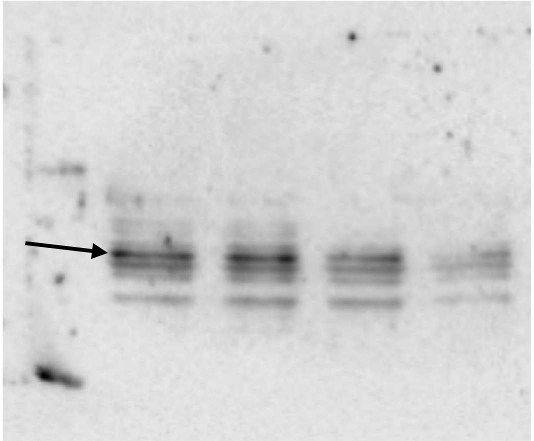

ISCU

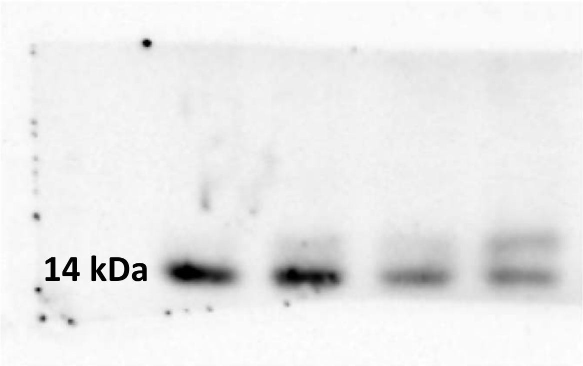

IRP1

98 kDa

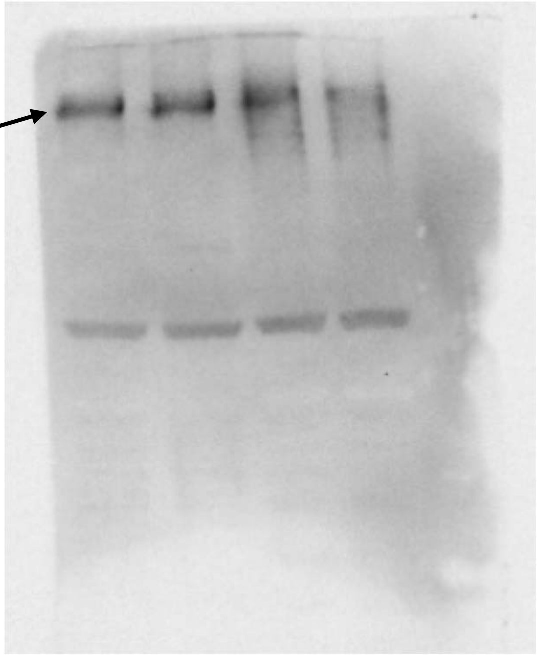

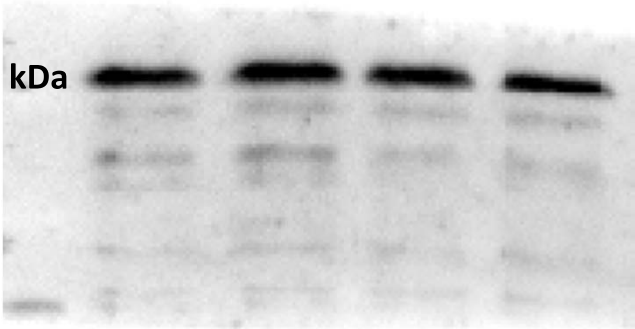

Actin

Uncropped gels of Supplementary Figure 13

MT-ND1

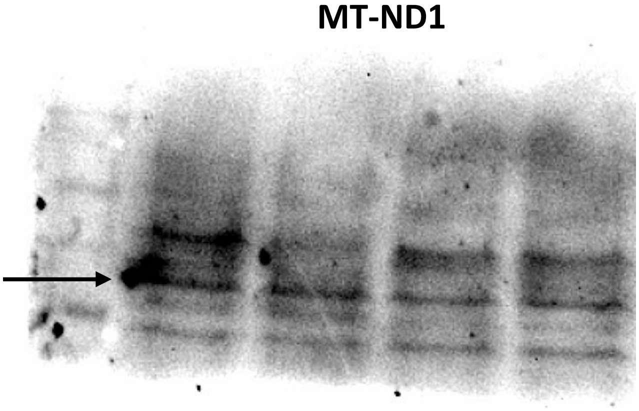

COX2

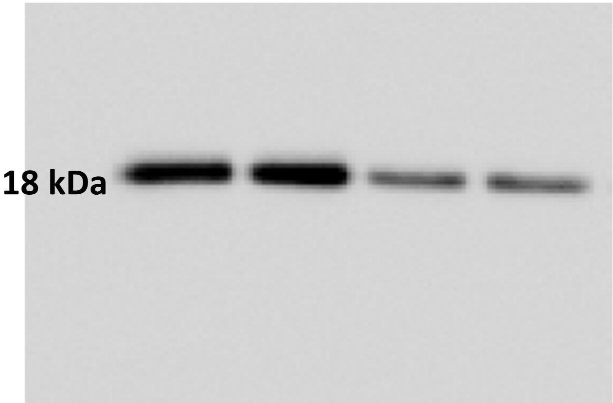

COX4

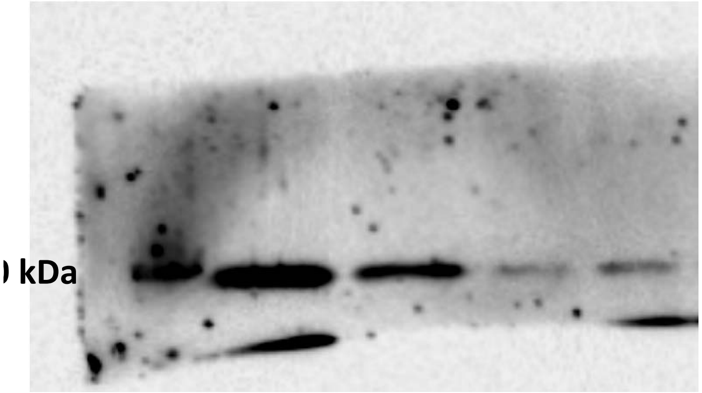

NDUFS4

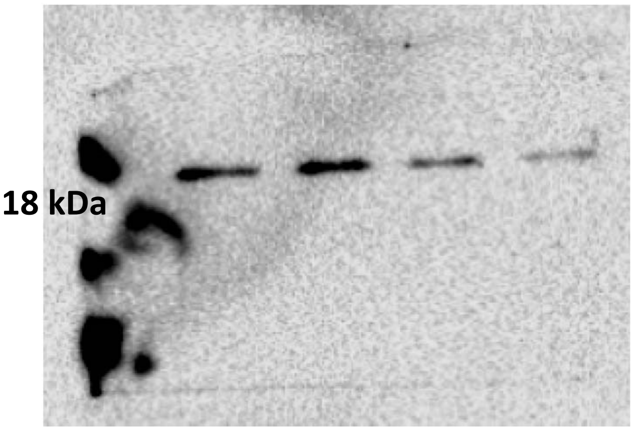

VDAC1

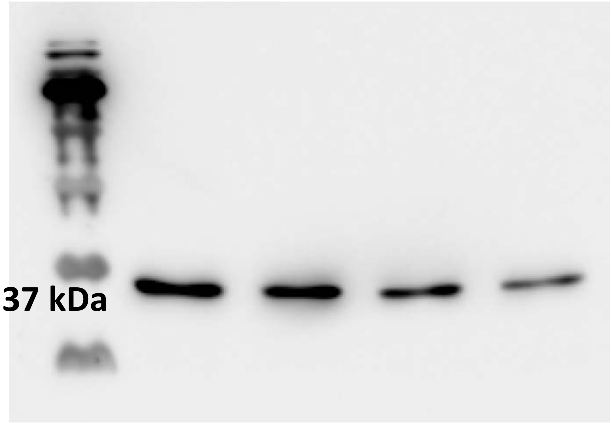

PGC1α

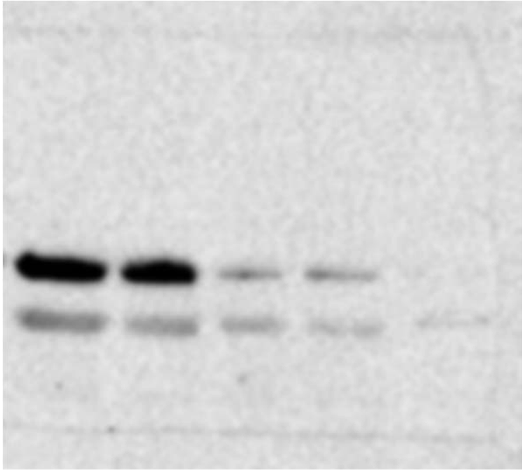

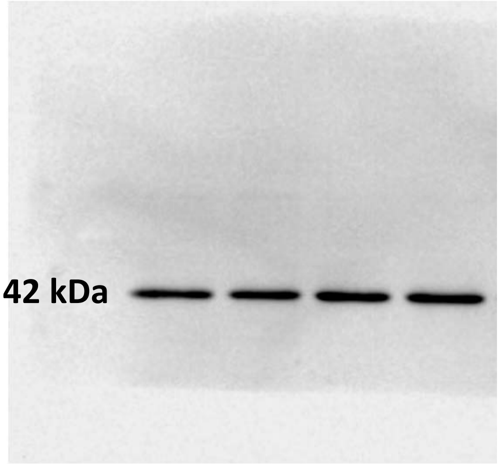

Actin

Uncropped gels of Supplementary Figure 8

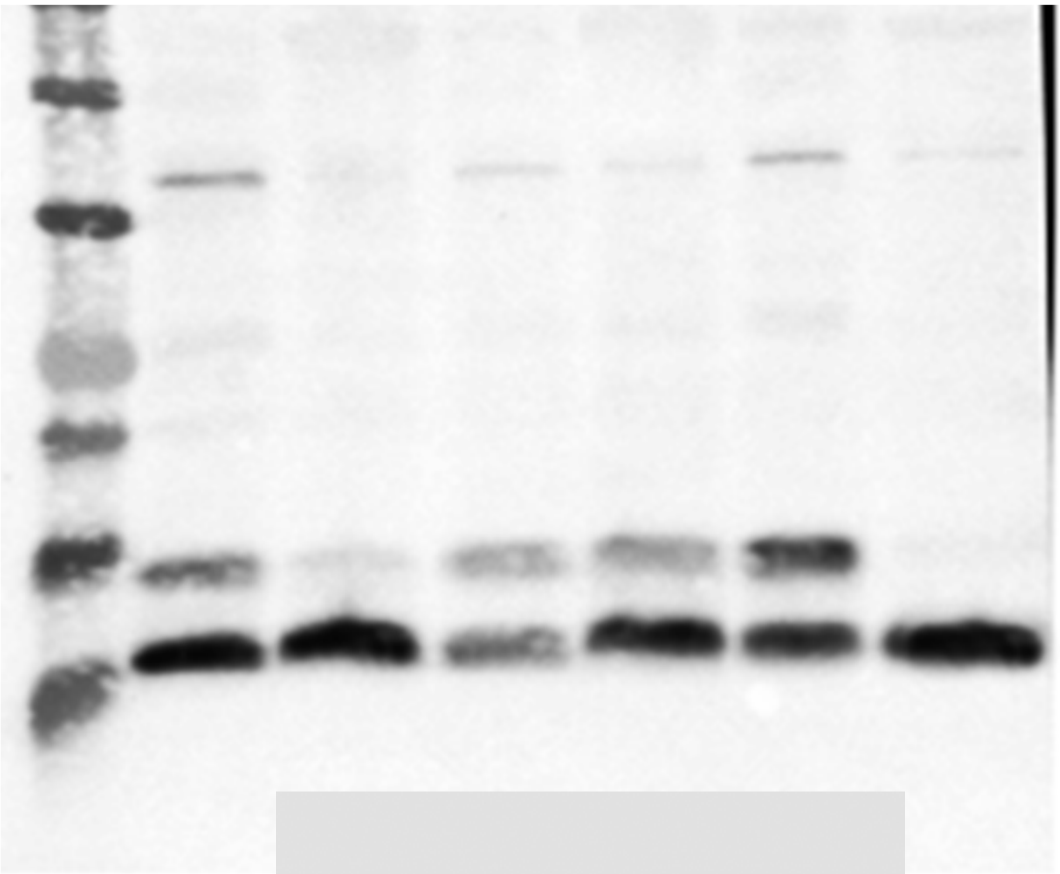

17 kDa LC3B-I

15 kDa LC3B-II

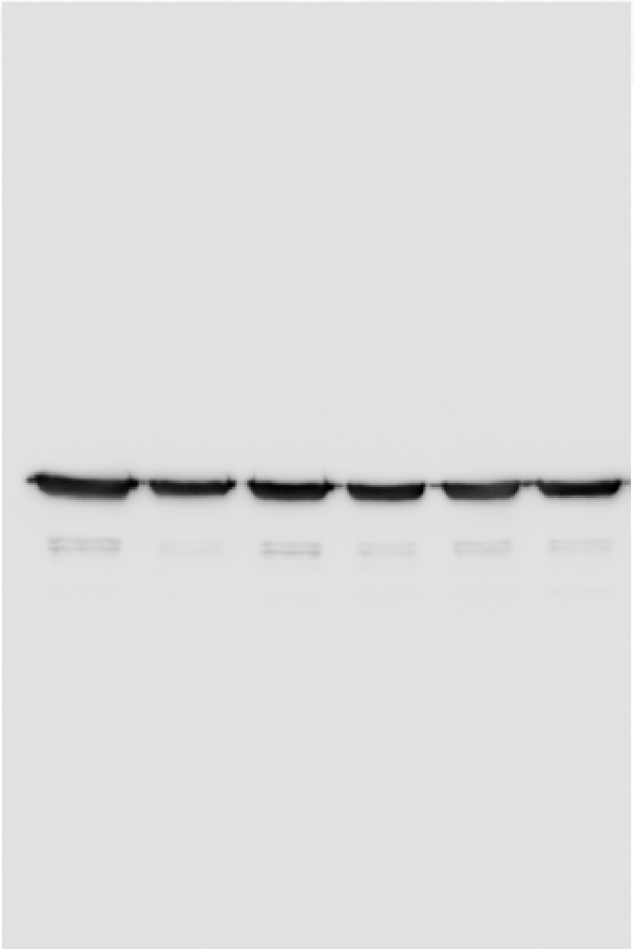

actina 42 kDa

Uncropped gels of Figure 13

WDR45

45 kDa

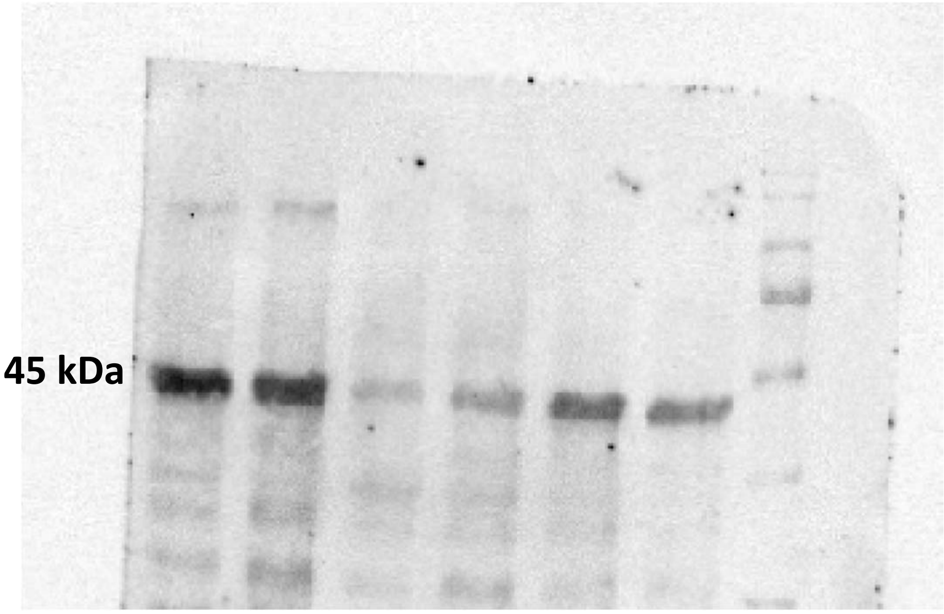

Actin

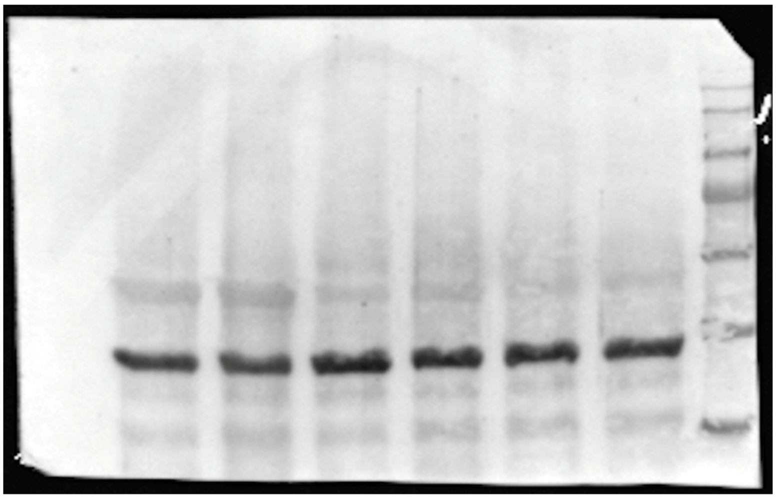

42 kDa
